# Risk factors, management, and outcomes of amniotic fluid embolism: A multicountry, population-based cohort and nested case-control study

**DOI:** 10.1371/journal.pmed.1002962

**Published:** 2019-11-12

**Authors:** Kathryn E. Fitzpatrick, Thomas van den Akker, Kitty W. M. Bloemenkamp, Catherine Deneux-Tharaux, Alexandra Kristufkova, Zhuoyang Li, Timme P. Schaap, Elizabeth A. Sullivan, Derek Tuffnell, Marian Knight

**Affiliations:** 1 National Perinatal Epidemiology Unit, Nuffield Department of Population Health, University of Oxford, Oxford, United Kingdom; 2 Department of Obstetrics and Gynaecology, Leiden University Medical Centre, Leiden, the Netherlands; 3 Birth Centre Wilhelmina Children Hospital, Division of Woman and Baby, University Medical Center Utrecht, Utrecht, the Netherlands; 4 Université de Paris, CRESS, Obstetrical Perinatal and Pediatric Epidemiology Research Team, EPOPé, INSERM, Paris, France; 5 First Department of Obstetrics and Gynaecology, Faculty of Medicine, Comenius University, Bratislava, Slovakia; 6 Australian Centre for Public and Population Health Research, Faculty of Health, University of Technology Sydney, Sydney, Australia; 7 Faculty of Health and Medicine, University of Newcastle, Callaghan, Australia; 8 Bradford Teaching Hospitals NHS Foundation Trust, Bradford, United Kingdom; University of Manchester, UNITED KINGDOM

## Abstract

**Background:**

Amniotic fluid embolism (AFE) remains one of the principal reported causes of direct maternal mortality in high-income countries. However, obtaining robust information about the condition is challenging because of its rarity and its difficulty to diagnose. This study aimed to pool data from multiple countries in order to describe risk factors, management, and outcomes of AFE and to explore the impact on the findings of considering United Kingdom, international, and United States AFE case definitions.

**Methods and findings:**

A population-based cohort and nested case-control study was conducted using the International Network of Obstetric Survey Systems (INOSS). Secondary data on women with AFE (*n* = 99–218, depending on case definition) collected prospectively in population-based studies conducted in Australia, France, the Netherlands, Slovakia, and the UK were pooled along with secondary data on a sample of control women (*n* = 4,938) collected in Australia and the UK. Risk factors for AFE were investigated by comparing the women with AFE in Australia and the UK with the control women identified in these countries using logistic regression. Factors associated with poor maternal outcomes (fatality and composite of fatality or permanent neurological injury) amongst women with AFE from each of the countries were investigated using logistic regression or Wilcoxon rank–sum test. The estimated incidence of AFE ranged from 0.8–1.8 per 100,000 maternities, and the proportion of women with AFE who died or had permanent neurological injury ranged from 30%–41%, depending on the case definition. However, applying different case definitions did not materially alter findings regarding risk factors for AFE and factors associated with poor maternal outcomes amongst women with AFE. Using the most liberal case definition (UK) and adjusting for the severity of presentation when appropriate, women who died were more likely than those who survived to present with cardiac arrest (89% versus 40%, adjusted odds ratio [aOR] 10.58, 95% confidence interval [CI] 3.93–28.48, *p* < 0.001) and less likely to have a source of concentrated fibrinogen (40% versus 56%, aOR 0.44, 95% CI 0.21–0.92, *p* = 0.029) or platelets given (24% versus 49%, aOR 0.23, 95% CI 0.10–0.52, *p* < 0.001). They also had a lower dose of tranexamic acid (median dose 0.7 g versus 2 g, *p* = 0.035) and were less likely to have had an obstetrician and/or anaesthetist present at the time of the AFE (61% versus 75%, aOR 0.38, 95% CI 0.16–0.90, *p* = 0.027). Limitations of the study include limited statistical power to examine factors associated with poor maternal outcome and the potential for residual confounding or confounding by indication.

**Conclusions:**

The findings of our study suggest that when an AFE is suspected, initial supportive obstetric care is important, but having an obstetrician and/or anaesthetist present at the time of the AFE event and use of interventions to correct coagulopathy, including the administration of an adequate dose of tranexamic acid, may be important to improve maternal outcome. Future research should focus on early detection of the coagulation deficiencies seen in AFE alongside the role of tranexamic acid and other coagulopathy management strategies.

## Abstract 2

Kathryn E. Fitzpatrick and colleagues report on risk factors and management of the rare condition amniotic fluid embolism.

## Author summary

**Why was this study done?:**

- Amniotic fluid embolism (AFE) is a rare pregnancy complication in which amniotic fluid or foetal material enters the woman’s blood stream and triggers a severe reaction.
- Women with AFE may collapse suddenly during or shortly after they give birth, and it often results in the death of the mother.
- The rarity of AFE, together with the fact that it is a hard condition to diagnose and several case definitions have been proposed, makes it difficult to obtain reliable information about the condition.

**What did the researchers do and find?:**

- Data on women with AFE (*n* = 99–218 depending on case definition) collected in five high-income countries were studied along with data on a sample of control women without AFE (*n* = 4,938) collected in two of the countries.
- Depending on the definition of AFE used, AFE was found to affect between 1 in every 125,000 to 1 in every 55,000 women giving birth, and between 30%–41% of women with the condition died or had permanent neurological injury.
- Applying different case definitions resulted in similar findings regarding factors associated with the occurrence of AFE and factors associated with poor maternal outcomes amongst women with AFE.
- Amongst women with AFE, those with poor maternal outcome were less likely to have had interventions such as platelets to correct blood clotting problems, had a lower dose of tranexamic acid, and were less likely to have had an obstetrician and/or anaesthetist present at the time of the AFE event.

**What do these findings mean?:**

- Our findings suggest that having an obstetrician and/or anaesthetist present at the time of the event and the use of interventions to correct blood clotting abnormalities, including the administration of an adequate dose of tranexamic acid, may be important to improve maternal outcomes in women with AFE.

## Introduction

Amniotic fluid embolism (AFE), although rare—affecting an estimated 1.7 per 100,000 maternities in the UK—remains one of the principal reported causes of direct maternal mortality in high-income countries [1–5]. The condition is characterised by unexplained sudden cardiovascular collapse, respiratory distress, and disseminated intravascular coagulation (DIC). Obtaining robust information about risk factors, management, and outcomes of AFE is challenging owing to the rarity of the condition, combined with the fact that clinical diagnosis of AFE is one of exclusion and various case definitions have been proposed [1,6–8]. Previous reviews have highlighted the lack of consistency in the factors reported to be associated with the occurrence of AFE and the limited evidence on factors associated with poor outcomes [2,9]. Analysis of pooled international data, obtained using consistent methodologies with agreed definitions, could provide more reliable information on these associated factors and hence provide the potential to develop appropriate evidence-based preventive strategies and guide best practice.

The International Network of Obstetric Survey Systems (INOSS) is a collaboration of organisations in over 15 countries conducting prospective population-based studies of uncommon and severe complications in pregnancy and childbirth using comparable surveillance systems [10]. Five INOSS members in Australia, France, the Netherlands, Slovakia, and the UK have collected data on women with AFE, and two of the INOSS members in Australia and the UK have also collected information on a sample of control women. The aim of this study was to pool these data in order to describe risk factors, management, and outcomes of AFE, as well as identify whether there are specific factors that are associated with poor maternal outcomes. We also aimed to explore the impact on the findings of considering UK, international, and US case definitions of AFE [1,6–8].

## Methods

### Study design and data collection

A multicountry, population-based cohort and nested case-control study was conducted using the INOSS. We pooled anonymous individual-level data on women with AFE that were collected prospectively in five population-based studies conducted by members of INOSS in Australia, France, the Netherlands, Slovakia, and the UK. The studies were conducted in Australia between 1 January 2010 and 31 December 2016, using the Australasian Maternity Outcomes Surveillance System (AMOSS) [11]; in France between 1 May 2012 and 30 November 2013, using the Épidémiologie de la Morbidité Maternelle Sévère (EPIMOMS) [12] study; in the Netherlands between 1 September 2013 and 31 August 2016, using the Netherlands Obstetric Surveillance System (NethOSS); in Slovakia between 1 January 2012 and 31 December 2016, using the Slovak Obstetric Survey System (SOSS); and in the UK between 1 February 2005 and 31 January 2018, using the UK Obstetric Surveillance System (UKOSS) [13]. Australia, Slovakia, and the UK have previously published some of their data in peer-reviewed journals [1,6,14–17]. With the exception of the French study, which was conducted in six regions of France (Alsace, Auvergne, Basse-Normandie, Ile-de-France, Lorraine, and Rhône-Alpes) covering one-fifth of all deliveries in the country, studies were nationwide, with nominated clinicians in each maternity unit in the country contacted on a monthly basis and asked to report all cases of AFE (see Table 1 for definitions of AFE used by participating countries). On reporting a case, clinicians were prompted to complete data collection forms from the woman’s medical notes to confirm the diagnosis and obtain additional information on potential risk factors, management (including coagulation and blood products, surgical interventions such as hysterectomy, and the presence of medical personnel at the time of the maternal collapse), and outcomes. In France, the completeness of case identification was checked through the review of delivery logbooks, hospital discharge data, and laboratory files. Maternal deaths reported through EPIMOMS were also cross-checked with maternal deaths reported to the National Confidential Enquiries on Maternal Mortality (ENCMM). In the Netherlands, maternal deaths from AFE reported through NethOSS were cross-checked with maternal deaths reported to the Netherlands Audit Committee Maternal Mortality and Morbidity. In Slovakia, maternal deaths from AFE reported through SOSS were cross-checked with mandatory reports of maternal deaths kept by the chief of Gynecology and Obstetrics of the Ministry of Health in Slovakia. In the UK, maternal deaths from AFE reported through UKOSS were cross-checked with maternal deaths reported to the Centre for Maternal and Child Enquiries (CMACE) and the National Maternal, Newborn and Infant Clinical Outcome Review Programme run by MBBRACE-UK.

**Table 1:** Case definitions of AFE.

	Criteria
**Definitions of AFE used by participating INOSS members**	
Australia (AMOSS)	Either as a clinical diagnosis (acute hypotension or cardiac arrest, acute hypoxia and coagulopathy in the absence of any other potential explanation for the symptoms and signs observed) or as a postmortem diagnosis (presence of foetal squames/debris in the pulmonary circulation).
France (EPIMOMS)	Women with SAMM attributed to AFE by the clinicians in charge. The EPIMOMS study used a standardised definition of SAMM that was developed through a national Delphi formal expert consensus process, intended to characterise maternal complications with severe health alteration and organ dysfunction. The multicriteria definition of SAMM combined diagnoses (severe obstetric bleeding, eclampsia, severe preeclampsia, pulmonary embolism, stroke, and psychiatric disorder), organ dysfunctions (hepatic, haematological, respiratory, cardiovascular, renal, and neurological), and interventions (admission to Intensive Care Unit and laparotomy after delivery).
Netherlands (NethOSS)	Same as UK (UKOSS).
Slovakia (SOSS)	Same as UK (UKOSS).
UK (UKOSS)	In the absence of any other clear cause, EITHER acute maternal collapse with one or more of the following features: Acute foetal compromise Cardiac arrest Cardiac rhythm problems Coagulopathy Hypotension Maternal haemorrhage Premonitory symptoms, e.g., restlessness, numbness, agitation, tingling Seizure Shortness of breath Excluding: women with maternal haemorrhage as the first presenting feature in whom there was no evidence of early coagulopathy or cardiorespiratory compromise OR Women in whom the diagnosis was made at postmortem examination with the finding of foetal squames or hair in the lungs.
**Other definitions of AFE**	
INOSS	An acute cardiorespiratory collapse within 6 hours after labour, birth, or ruptured membranes, with no other identifiable cause, followed by acute coagulopathy in those women who survive the initial event.
Clark and colleagues [8]	1. Sudden onset of cardiorespiratory arrest, or both hypotension (systolic blood pressure <90 mm Hg) and respiratory compromise (dyspnoea, cyanosis, or peripheral capillary oxygen saturation [SpO2] <90%). 2. Documentation of overt DIC following appearance of these initial signs or symptoms, using scoring system of Scientific and Standardization Committee on DIC of the ISTH, modified for pregnancy.* Coagulopathy must be detected prior to loss of sufficient blood to itself account for dilutional or shock-related consumptive coagulopathy. 3. Clinical onset during labour or within 30 minutes of delivery of placenta. 4. No fever (≥38.0 °C) during labour.

*Scoring system of Scientific and Standardization Committee on DIC of the ISTH, modified for pregnancy:
Platelet count: > 100,000/mL = 0, <100,000/mL = 1, <50,000/mL = 2; Prolonged prothrombin time or international normalized ratio: <25% increase = 0, 25%–50% increase = 1, >50% increase = 2; Fibrinogen level: >200 mg/L = 0, <200 mg/L = 1.

Score ≥ 3 is compatible with overt DIC in pregnancy

**Abbreviations**: AFE, amniotic fluid embolism; AMOSS, Australasian Maternity Outcomes Surveillance System; DIC, disseminated intravascular coagulation; EPIMOMS, Épidémiologie de la Morbidité Maternelle Sévère; INOSS, International Network of Obstetric Survey Systems; ISTH, International Society on Thrombosis and Hemostasis; NethOSS, Netherlands Obstetric Surveillance System; SAMM, severe acute maternal morbidity; SOSS, Slovak Obstetric Survey System; UKOSS, UK Obstetric Surveillance System.

We also pooled anonymous individual-level data on a sample of control women without AFE that were collected in Australia and the UK. In Australia, the control women were identified by AMOSS reporting clinicians as the two women delivering immediately before other AMOSS study cases identified between January 2010 and December 2011 [18]. The AMOSS reporting clinicians were asked to complete a data collection form for the control women that was identical to that used to capture general non-case–specific information about all conditions, including AFE, studied by AMOSS. In the UK, the control women were identified by UKOSS reporting clinicians as the two women delivering in the same hospital immediately before other UKOSS study cases identified at various periods between February 2005 and June 2014 [19–27]. The UKOSS reporting clinicians were asked to complete a data collection form for the control women that was identical to that used for the particular UKOSS study cases, with the exception of the case-specific information. Although UKOSS individually develops data collection forms for each condition it studies, the data collection forms for the different conditions contain common data items about women’s characteristics. The control women were comparable in characteristic to the available national data on women giving birth in the respective countries [28–30].

### Case definition

For the purpose of this study, three case definitions of AFE were considered: 1) the UKOSS case definition [1,6]; 2) a definition of AFE developed through a Delphi process by members of INOSS [7], hereafter referred to as the INOSS case definition; and 3) a definition of AFE proposed by Clark and colleagues [8], hereafter referred to as the Clark case definition (see Table 1 for details of each of these case definitions). Because of the data available in each of the participating INOSS countries, we had to apply the following modifications to the INOSS and Clark criteria. Women were identified as having acute cardiorespiratory collapse, a component of the INOSS case definition, if they had any of the following features present at or immediately preceding diagnosis defined according to usual hospital ranges: cardiac arrest, cardiac rhythm problems, or hypotension. Women were considered as having the first criterion of the Clark case definition (sudden onset of cardiorespiratory arrest or both hypotension and respiratory compromise) if they had the following features present at or immediately preceding diagnosis: cardiac arrest or cardiac rhythm problems or both hypotension and shortness of breath. Because only a few women in our study had sufficient laboratory results collected to determine if they met the second criterion of the Clark definition (overt DIC; see Table 1), women were considered to meet this criterion or the acute coagulopathy component of the INOSS definition if they had coagulopathy recorded as a feature present at or immediately preceding diagnosis or had the laboratory levels compatible with DIC that feature in the Clark definition. Because none of the participating INOSS countries collected data on the time the placenta was delivered, women were determined as meeting the third criterion of the Clark case definition (clinical onset during labour or within 30 minutes of delivery of placenta) if they had clinical onset during labour or within 60 minutes of delivery. Finally, women were considered as meeting the fourth criterion of the Clark case definition (no fever [≥38.0 °C] during labour) if they had no evidence of infection recorded or had an infection that was clearly prelabour or postevent. Recognising that we applied these modifications to the INOSS and Clark criteria, we hereafter refer to these definitions in our study as modified INOSS and Clark case definitions, respectively.

### Statistical analysis

All analyses were prespecified as described in detail in the methods section and outlined in the study protocol (S1 Study Protocol), with the exception of adjusting, when possible, the analysis of maternal outcome in relation to the timing of when interventions were used for cardiac arrest. This was performed in response to peer reviewer comments. As stated in the study protocol, we had originally planned to include AFE cases collected in New Zealand and Denmark but did not include these cases because data sharing with INOSS members in these countries was not possible in the timeline of this study.

Incidence rates for AFE with exact Poisson 95% confidence intervals (95% CIs) were calculated using as a denominator the available data on the number of maternities recorded regionally or nationally during each study period. Putative risk factors for AFE identified from the literature were investigated by comparing the women with AFE in the UK and Australia to the control group of women identified in these countries using unconditional logistic regression to estimate odds ratios (ORs) and 95% CIs. A full regression model was developed by including both explanatory and potential confounding factors in a core model if there was a pre-existing hypothesis or evidence to indicate that they were causally related to AFE. Plausible interactions, including whether there was evidence to suggest that any associations varied by country, were assessed by the addition of interaction terms to the full model and subsequent likelihood ratio testing. Using fractional polynomials, there was no evidence that continuous variables showed evidence of departure from linearity. Continuous variables were therefore treated as continuous linear terms when adjusting for them in the analysis but were presented as categorical variables for ease of interpretation.

The pooled data on women with AFE from each of the five countries were used to describe the management and outcomes of AFE. Factors associated with poor maternal outcomes (fatality and composite of fatality or permanent neurological injury, including persistent vegetative state, anoxic/hypoxic brain injury, or cerebrovascular accident, with the composite hereafter referred to as severe outcome) amongst women with AFE from each of the countries were investigated using unconditional logistic regression or Wilcoxon rank–sum test as appropriate. In all analyses, only factors that were collected in a comparable way between countries were included. Because the proportion of missing data was low for most variables of interest and mostly missing because of certain data items not being collected in one or more of the countries (see table footnotes), only the observed data were analysed (complete case analysis). All *p*-values were two-sided with the significance level set at <0.05, except for interaction tests, in which the significance level was set at <0.01 to allow for multiple testing. All analyses were performed using STATA v. 15 (StataCorp, College Station, TX, USA). This study is reported as per the Strengthening the Reporting of Observational Studies in Epidemiology (STROBE) guideline (S1 STROBE Checklist).

### Sample size and power

The sample size was predetermined by the sample size of the participating countries existing studies. Assuming putative risk factors have a prevalence of 5% or 40%, the AFE risk factor analysis had 80% power at the 5% level of significance to detect ORs of 2.2 or greater and 1.6 or greater, respectively, using the UKOSS case definition; 2.5 or greater and 1.7 or greater, respectively, using the modified INOSS case definition; and 2.9 or greater and 1.9 or greater, respectively, using the modified Clark case definition. Assuming factors assessed have a prevalence of 5% or 40%, the analysis examining factors associated with fatality amongst AFE cases had 80% power at the 5% significance level to detect ORs of 5.0 or greater and 2.8 or greater, respectively, using the UKOSS case definition; 5.9 or greater and 3.1 or greater, respectively, using the modified INOSS case definition; and 8.4 or greater and 4.5 or greater, respectively, using the modified Clark case definition. The corresponding figures for the analysis of factors associated with death or permanent neurological injury were an OR of 4.8 or greater and 2.6 or greater, respectively, using the UKOSS case definition; 6.1 or greater and 3.1 or greater, respectively, using the modified INOSS case definition; and 8.4 or greater and 4.2 or greater, respectively, using the modified Clark case definition.

### Approvals

This was a secondary analysis of previously collected anonymous data. The only ethical approval required was to export data from Australia to the UK for the analysis of this study. Ethical approval (reference: HREC/09/CIPHS/21) was granted by the New South Wales Population and Health Services Research Ethics Committee [31]. Data sharing agreements were also signed with each participating institution.

## Results

**Fig 1:**
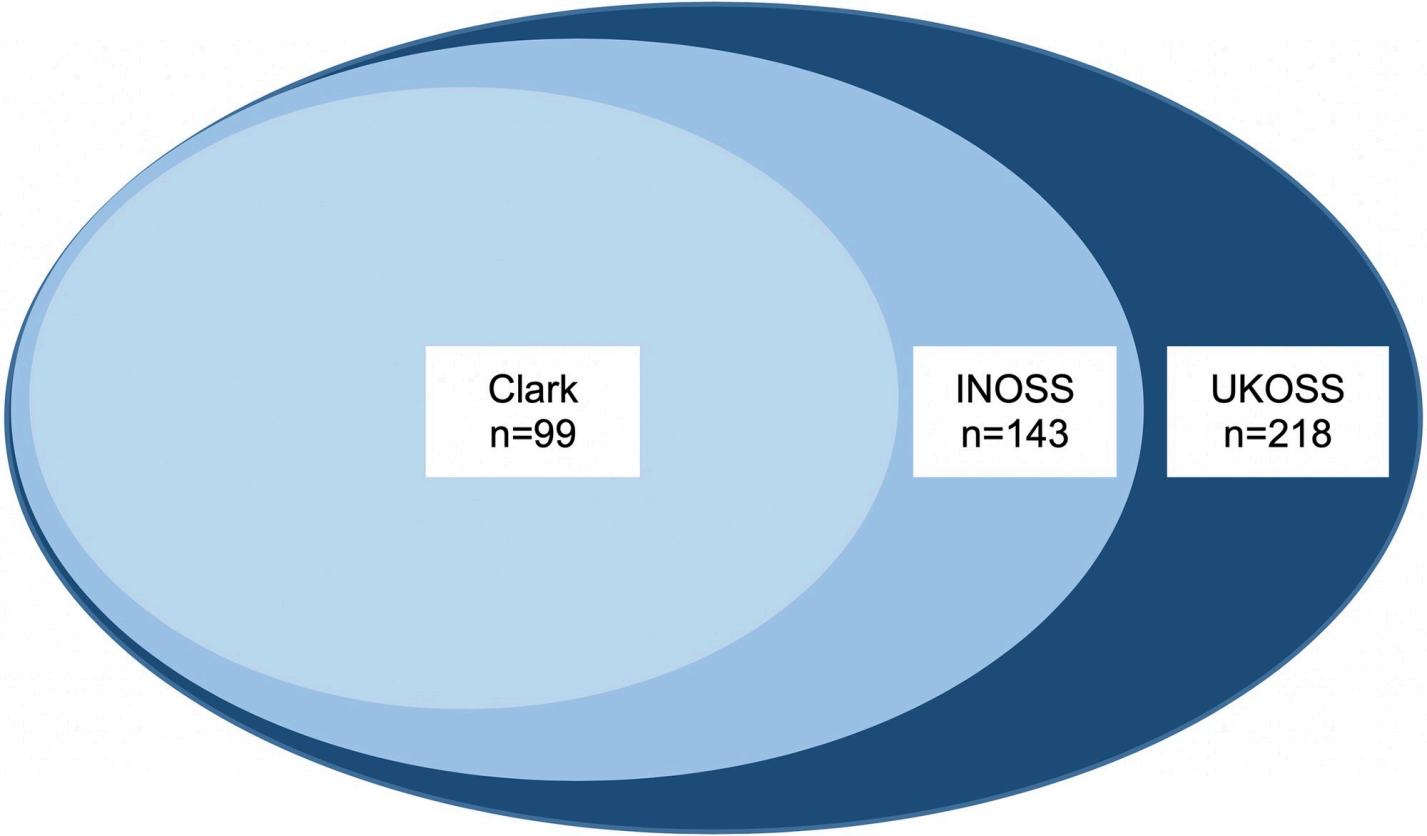
Total number of women in Australia, France, the Netherlands, Slovakia, and the UK meeting UKOSS case definition (dark blue), modified INOSS case definition (medium blue), and modified Clark case definition (light blue) of AFE. AFE, amniotic fluid embolism; INOSS, International Network of Obstetric Survey Systems; UKOSS, UK Obstetric Surveillance System.

Table 2 shows the number and incidence of AFE by definition of AFE used and by country, and Fig 1 illustrates the number of women meeting each case definition of AFE. The incidence of AFE varied according to the definition of AFE applied with the overall incidence 1.8 per 100,000 maternities (95% CI 1.5–2.0) using the UKOSS case definition; 1.2 per 100,000 maternities (95% CI 1.0–1.4) using the modified INOSS case definition; and 0.8 per 100,000 maternities (95% CI 0.6–1.0) using the modified Clark case definition. A lack of evidence of coagulopathy/DIC was the most common reason for the UKOSS cases not meeting the other case definitions, with 43 women and 36 women not meeting the modified INOSS and Clark case definitions, respectively, on this one criterion alone. The apparently higher incidence of AFE in France compared to the other countries when the UKOSS case definition was used was not evident when the modified Clark case definition was applied. Of the six women in France who met the UKOSS but not the modified Clark case definition, two women did not meet the modified Clark definition because it could not be determined whether they had clinical onset during labour or within 60 minutes of delivery because of missing data; two of the women did not meet the modified Clark definition because they did not have clinical onset during labour or within 60 minutes of delivery, one of whom additionally had no evidence of coagulopathy/DIC; and the remaining two women did not meet the modified Clark definition because they did not have evidence of cardiorespiratory arrest or both hypotension and respiratory compromise.

**Table 2:** Numbers and incidence of AFE (95% CI) per 100,000 maternities^¥^ by definition of AFE and by country.

Country	Study Period	Total No. of Maternities^¥^ during Study Period	Definition Used					
			UKOSS		Modified INOSS		Modified Clark	
			No. of Cases	Incidence (95% CI)	No. of Cases	Incidence (95% CI)	No. of Cases	Incidence (95% CI)
**Australia**	01/01/2010–31/12/2016	1,959,945	36	1.8 (1.3–2.5)	28	1.4 (0.9–2.1)	20	1.0 (0.6–1.6)
**France**	01/05/2012–30/11/2013	182,309	8	4.4 (1.9–8.7)	4	2.2 (0.6–5.6)	2	1.1 (0.1–4.0)
**Netherlands**	01/09/2013–31/08/2016	502 559	8	1.6 (0.7–3.1)	5	1.0 (0.3–2.3)	2	0.4 (0.05–1.4)
**Slovakia**	01/01/2012–31/12/2016	276,098	5	1.8 (0.6–4.2)	3	1.1 (0.2–3.2)	3	1.1 (0.2–3.2)
**UK**	01/02/2005–31/01/2018	9,986,092	161	1.6 (1.4–1.9)	103	1.0 (0.8–1.3)	72	0.7 (0.6–0.9)
**TOTAL**		12,404,444	218	1.8 (1.5–2.0)	143	1.2 (1.0–1.4)	99	0.8 (0.6–1.0)

^¥^Australia: ≥20 week gestation or resulting in birth of a baby weighting ≥400 g; France: ≥22 weeks gestation; Netherlands, Slovakia, and UK: ≥24 weeks gestation.

**Abbreviations**: AFE, amniotic fluid embolism; CI, confidence interval; INOSS, International Network of Obstetric Survey Systems; no., number; UKOSS, UK Obstetric Surveillance System.

### Risk factors

Table 3 shows the characteristics of the women in the UK and Australia with AFE according to the UKOSS case definition compared to the control women identified in these countries. The odds of having AFE rose significantly with increasing maternal age, showing no evidence of departure from linearity (adjusted OR [aOR] 1.12, 95% CI 1.08–1.15, *p* < 0.001 for every 1-year increase in age; presented in Table 3 as a binary variable for ease of interpretation). The odds of having AFE were also significantly raised in women who had a multiple pregnancy, polyhydramnios, placenta praevia, placental abruption, and induction of labour using any method. Information on prostaglandin labour induction and oxytocin use in labour was available for the women with AFE and a subset of the control women. Adjusted analysis revealed that the odds of having AFE were significantly raised in women induced with prostaglandin compared to those induced without prostaglandin (aOR 2.46, 95% CI 1.17–5.15, *p* = 0.017) but were not significantly higher in women who had oxytocin used during labour compared to those who laboured without oxytocin (aOR 1.31, 95% CI 0.83–2.07, *p* = 0.240).

**Table 3:** Comparison of characteristics of women with AFE according to UKOSS case definition and control women in the UK and Australia.

	Number (%)^a^ of Cases (*n* = 197)	Number (%)^a^ of Controls (*n* = 4,938)	Unadjusted OR (95% CI, *p*-Value)	aOR (95% CI, *p*-Value)^Ϯ^
**Sociodemographic characteristics**				
Maternal age (years)^¥^				
Less than 35	112 (57)	3,884 (79)	1	1
35 or more	85 (43)	1,048 (21)	**2.81 (2.10–3.76, <0.001)**	**2.40 (1.71–3.37, <0.001)**
Body mass index at booking (kg/m^2^)^¥^				
Less than 30	143 (79)	3,677 (80)	1	1
30 or more	39 (21)	892 (20)	1.12 (0.78–1.61, 0.526)	1.05 (0.70–1.58, 0.818)
Smoking status^¥^				
Never/ex-smoker	168 (87)	3,979 (82)	1	1
Smoked during pregnancy	26 (13)	860 (18)	0.72 (0.47–1.09, 0.119)	1.20 (0.74–1.92, 0.458)
**Previous obstetric and medical history**				
Parity^¥^^,^^b^				
0	77 (−40)	2,134 (43)	1	1
1 or more	117 (60)	2,797 (57)	1.16 (0.86–1.55, 0.323)	0.90 (0.64–1.26, 0.522)
Chronic hypertension^¥^				
No	186 (95)	4,862 (99)	1	1
Yes	9 (5)	48 (1)	**4.90 (2.08–10.29, <0.001)**	1.10 (0.37–3.29, 0.867)
Pre-existing diabetes^¥^				
No	194 (99)	4,860 (99)	1	
Yes	2 (1)	51 (1)	0.98 (0.12–3.78, 1.000)	
**Current pregnancy characteristics**				
Multiple pregnancy				
No	178 (90)	4,866 (99)	1	1
Yes	19 (10)	71 (1)	**7.31 (4.07–12.59, <0.001)**	**6.28 (3.23–12.22, <0.001)**
Gestational diabetes^¥^				
No	183 (93)	4,742 (97)	1	1
Yes	13 (7)	160 (3)	**2.11 (1.17–3.78, 0.013)**	1.44 (0.74–2.79, 0.284)
Hypertensive disorder^¥^				
No	174 (89)	4,688 (96)	1	1
Yes	22 (11)	215 (4)	**2.76 (1.73–4.39, <0.001)**	1.48 (0.79–2.75, 0.217)
Polyhydramnios^¥^				
No	188 (96)	4,866 (99)	1	1
Yes	8 (4)	32 (1)	**6.47 (2.54–14.58, <0.001)**	**5.04 (1.90–13.36, 0.001)**
Placenta praevia^¥^				
No	180 (92)	4,878 (99)	1	1
Yes	16 (8)	31 (1)	**13.96 (7.00–26.88, <0.001)**	**13.26 (6.35–27.69, <0.001)**
Placental abruption^¥^				
No	191 (97)	4,893 (100)	1	1
Yes	5 (3)	8 (0.2)	**15.98 (4.07–56.01, <0.001)**	**14.14 (3.52–56.86, <0.001)**
Induction of labour using any method^¥^^,^^c^				
No	117 (59)	3,752 (76)	1	1
Yes	80 (41)	1,179 (24)	**2.18 (1.63–2.91, <0.001)**	**2.37 (1.68–3.34, <0.001)**
Gestational age at delivery (weeks)^¥^^,^^d^				
Term (37–41)	160 (82)	4,412 (90)	1	1
Preterm (<37)	31 (16)	369 (8)	**2.32 (1.55–3.45, <0.001)**	1.22 (0.72–2.09, 0.462)
Post-term (42 or more)	3 (2)	136 (3)	0.61 (0.19–1.93, 0.399)	0.58 (0.18–1.89, 0.366)
Macrosomia (birthweight of 4,000 g or more)^¥^^,^^d^				
No	169 (90)	4,359 (89)	1	1
Yes	19 (10)	558 (11)	0.88 (0.54–1.42, 0.598)	0.87 (0.51–1.48, 0.605)

^a^Percentage of those with complete data.

^Ϯ^Adjusted for all variables in table apart from pre-existing diabetes.

^b^Australia (AMOSS): number of previous pregnancies ≥20 week gestation or resulting in birth of a baby weighting ≥400g; UK (UKOSS): number of completed pregnancies ≥24 week gestation.

^c^In Australia, data on induction of labour only collected for women who laboured. Women who did not labour in Australia assumed to have had no induction of labour.

^d^Excludes 11 women who had pregnancies ending before 24 week gestation

^¥^Missing data: maternal age *n* = 6, 0.1%; body mass index *n* = 384, 7.5%; smoking status *n* = 102, 2.0%; parity *n* = 10, 0.2%; chronic hypertension *n* = 30, 0.6%; pre-existing diabetes *n* = 28, 0.5%; multiple pregnancy *n* = 1, 0.02%; gestational diabetes *n* = 37, 0.7%; hypertensive disorder *n* = 36, 0.7%; polyhydramnios *n* = 41, 0.8%; placenta praevia *n* = 30, 0.6%; placental abruption *n* = 38, 0.7%; labour induced *n* = 7, 0.1%; gestational age at delivery *n* = 13, 0.3%; macrosomia *n* = 19, 0.4%.

Bold text indicates statistically significant findings at the 5% level.

**Abbreviations**: AFE, amniotic fluid embolism; AMOSS, Australasian Maternity Outcomes Surveillance System; aOR, adjusted OR; CI, confidence interval; OR, odds ratio; UKOSS, UK Obstetric Surveillance System.

Considering just the AFE cases that occurred postnatally, both instrumental vaginal birth and cesarean section were associated with significantly raised odds of having AFE postnatally (aOR 9.82, 95% CI 3.93–24.49, *p* < 0.001 and aOR 14.02, 95% CI 6.25–31.42, *p* < 0.001, respectively). Of those who had AFE after a cesarean section, 61% (41/67) did not labour, and 81% (54/67) had the urgency of cesarean section recorded, with cesarean performed as an emergency (grade or category 1 or 2 urgency [32,33]) in 22 of the women (12 for foetal compromise, 5 for maternal compromise, 5 for other indications) and as an elective procedure (grade or category 3 or 4 urgency [32,33]) in 32 of the women (10 for previous cesarean birth, 4 for maternal request, 5 for abnormal presentation, and 13 for other indications). No significant interactions were found, and the nature of the associations was unaffected by adjusting for country.

Applying the modified INOSS or modified Clark case definition (S1 and S2 Tables) did not change the findings, with the exceptions of gestational diabetes, placental abruption, and instrumental vaginal birth, when the modified Clark definition was applied. Using the modified Clark case definition, women with gestational diabetes had significantly raised odds of having AFE (aOR 2.22, 95% CI 1.00–4.91, *p* = 0.049), while those with placental abruption and instrumental vaginal birth no longer had significantly higher odds of having the condition (aOR 4.70, 95% CI 0.46–47.59, *p* = 0.190 and aOR 2.97, 95% CI 0.63–13.88, *p* = 0.167, respectively), noting the more limited power of the analysis and the borderline significance of the gestational diabetes finding.

### Presentation, management, and outcomes of AFE

The proportion of women with AFE who died and the proportion who had a severe outcome (died or had permanent neurological injury) varied slightly according to the definition of AFE used: using the UKOSS case definition, 21% (45/216, 95% CI 16%–27%) of all women with AFE died, and 30% (57/193, 95% CI 23%–37%) had a severe outcome; using the modified INOSS case definition, 30% (42/142, 95% CI 22%–38%) of all women with AFE died, and 41% (51/125, 95% CI 32%–50%) had a severe outcome; and using the modified Clark case definition, 24% (24/99, 95% CI 16%–34%) of all women with AFE died, and 39% (33/84, 95% CI 29%–51%) had a severe outcome.

Table 4 shows the presentation and haematological parameters of women with AFE according to the UKOSS case definition by outcome. Applying the UKOSS case definition, women with AFE who had a severe outcome were more likely than those who did not have a severe outcome to present with cardiac arrest. This finding was also apparent when the analysis was limited to comparing women with AFE who died with those who survived (Table 4) and when the modified INOSS or modified Clark case definition was applied (S3 and S4 Tables). In the adjusted analysis, no other significant difference in presentation and haematological parameters were apparent between the women who had poor outcome and those who did not, except when the modified INOSS case definition was applied and the analysis limited to comparing women who died to those who survived. When the modified INOSS case definition was used, women who died were also found to be less likely than those who survived to have coagulopathy noted at presentation (aOR 0.16, 95% CI 0.04–0.64, *p* = 0.009), having adjusted for other factors that were significant in the unadjusted model, including cardiac arrest at presentation (S3 Table). Considering just the women with AFE according to the UKOSS case definition who did not present with cardiac arrest, 47% (48/103) presented at or before delivery, with 42% (45/108) noted to have acute fatal compromise at presentation, 20% (21/106) noted to have cardiac rhythm problems at presentation, 60% (65/108) noted to have coagulopathy at presentation, 77% (83/108) noted to have hypotension at presentation, 67% (72/108) noted to have maternal haemorrhage at presentation, 47% (49/104) noted to have premonitory symptoms at presentation, 14% (15/106) noted to have seizures at presentation, and 56% (59/106) noted to have shortness of breath at presentation.

**Table 4:** Using the UKOSS case definition, comparison of the presentation and haematological parameters of AFE cases who died to those who survived and of AFE cases that had severe outcome to those that did not have severe outcome.

	No. (%)^a^ of Cases that Died (*n* = 45)	No. (%)^a^ of Cases that Survived (*n* = 171)	Unadjusted OR (95% CI, *p*-Value)	aOR (95% CI, *p*-Value)^Ϯ^	No. (%)^a^ of Cases that Had Severe Outcome^b^ (*n* = 57)^c^	No. (%)^a^ of Cases that Did Not Have Severe Outcome^b^ (*n* = 136)^c^	Unadjusted OR (95% CI, *p*-Value)	aOR (95% CI, *p*-Value)^Ϯ^
**When AFE occurred**^¥^								
Before or at delivery	23 (55)	77 (47)	1		29 (55)	64 (48)	1	
Postdelivery	19 (45)	88 (53)	0.72 (0.37–1.43, 0.350)		24 (45)	68 (52)	0.78 (0.41–1.48, 0.444)	
**Features at presentation**^‡^								
Acute foetal compromise	15 (33)	62 (36)	0.88 (0.44–1.76, 0.716)		20 (35)	49 (36)	0.96 (0.50–1.83, 0.901)	
Cardiac arrest	40 (89)	68 (40)	**12.12 (4.55–32.25, <0.001)**	**10.58 (3.93–28.48, <0.001)**	50 (88)	49 (36)	**12.68 (5.34–30.12, <0.001)**	**11.50 (4.17–31.98, <0.001)**
Cardiac rhythm problems^¥^	9 (24)	45 (27)	0.83 (0.36–1.88, 0.652)		11 (23)	35 (26)	0.83 (0.38–1.81, 0.643)	
Coagulopathy	27 (60)	108 (63)	0.87 (0.45–1.71, 0.697)		35 (61)	78 (57)	1.18 (0.63–2.23, 0.603)	
Hypotension^¥^	21 (48)	120 (70)	**0.39 (0.20–0.76, 0.006)**	0.57 (0.27–1.19, 0.134)	28 (50)	94 (69)	**0.45 (0.24–0.85, 0.013)**	0.55 (0.24–1.23, 0.144)
Maternal haemorrhage	31 (69)	118 (69)	0.99 (0.49–2.02, 0.988)		38 (67)	90 (66)	1.02 (0.53–1.97, 0.948)	
Premonitory symptoms^¥^	21 (51)	69 (43)	1.38 (0.70–2.75, 0.354)		26 (51)	52 (39)	1.60 (0.83–3.07, 0.157)	
Seizures^¥^	7 (16)	24 (14)	1.14 (0.45–2.84, 0.786)		9 (16)	19 (14)	1.17 (0.49–2.77, 0.723)	
Shortness of breath^¥^	14 (32)	73 (44)	0.59 (0.29–1.20, 0.148)		17 (30)	60 (44)	0.54 (0.28–1.06, 0.073)	
**Platelet count (mL)**^¥^								
More than 100,000	10 (29)	61 (38)	1		13 (28)	49 (39)	1	1
50,000–100,000	11 (31)	65 (40)	1.03 (0.41–2.60), 0.946		15 (32)	54 (43)	1.05 (0.45–2.42, 0.914)	1.00 (0.39–2.57, 0.994)
Less than 50,000	14 (40)	35 (22)	2.44 (0.98–6.07), 0.055		19 (40)	24 (19)	**2.98 (1.27–7.04, 0.013)**	1.89 (0.70–5.10, 0.208)
**INR**^¥^								
<25% increase	2 (25)	13 (28)	1		2 (20)	10 (40)	1	
25%–50% increase	1 (13)	10 (21)	0.66 (0.01–14.43, 1.000)		2 (20)	7 (28)	1.40 (0.08–23.88, 1.000)	
>50% increase	5 (63)	24 (51)	1.35 (0.19–16.00, 1.000)		6 (60)	8 (32)	3.56 (0.46–45.58, 0.310)	
**Fibrinogen (g/L)**^¥^								
2 or more	0 (0)	12 (21)	1		2 (15)	7 (23)	1	
Less than 2	8 (100)	46 (79)	2.74 (0.38–infinity, 0.362)		11 (85)	24 (77)	1.59 (0.24–18.11, 0.925)	

^a^Percentage of those with complete data.

^b^Died or had permanent neurological injury.

^c^Data on maternal morbidity only collected from 2014 in Australia.

^Ϯ^Adjusted for factors that were significant (*p* < 0.05) in unadjusted model.

^‡^Features at presentation are not mutually exclusive,

^¥^Missing data/data not collected: when AFE occurred *n* = 9, 4.2% in died versus survived analysis and *n* = 8, 4.1% in severe outcome versus no severe outcome analysis; cardiac rhythm problems *n* = 13, 6.0% in died versus survived analysis and *n* = 12, 6.2% in severe outcome versus no severe outcome analysis (not collected in France); hypotension *n* = 1, 0.5%; premonitory symptoms *n* = 15, 6.9% in died versus survived analysis and *n* = 10, 5.2% in severe outcome versus no severe outcome analysis (not collected in France); seizures *n* = 4, 1.9% in died versus survived analysis and *n* = 2, 1.0% in severe outcome versus no severe outcome analysis; shortness of breath *n* = 6, 2.8% in died versus survived analysis and *n* = 2, 1.0% in severe outcome versus no severe outcome analysis; platelet count *n* = 20, 9.3% in died versus survived analysis and *n* = 19, 9.8% in severe outcome versus no severe outcome analysis; INR *n* = 161, 74.5% in died versus survived analysis and *n* = 158, 81.9% in severe outcome versus no severe outcome analysis (not collected in the UK before February 2015 and not collected in France); fibrinogen *n* = 150, 69.4% in died versus survived analysis and *n* = 149, 77.2% in severe outcome versus no severe outcome analysis (not collected in the UK before February 2015).

Bold text indicates statistically significant findings at the 5% level.

**Abbreviations**: AFE, amniotic fluid embolism; aOR, adjusted OR; CI, confidence interval; INR, International Normalized Ratio; OR, odds ratio; UKOSS, UK Obstetric Surveillance System.

In terms of sociodemographic, previous obstetric, medical history, and current pregnancy characteristics, women with AFE according to the UKOSS case definition who had a severe outcome only differed from those who did not have a severe outcome in that they were more likely to be multiparous (OR 2.00, 95% CI 1.01–3.95, *p* = 0.047, S5 Table). However, this association was no longer statistically significant having adjusted for cardiac arrest at presentation as a marker of the severity of presentation (aOR 1.91, 95% CI 0.89–4.08, *p* = 0.097). No significant differences in characteristic were apparent when the analysis was limited to comparing women who died to those who survived (S5 Table). In addition, no significant differences in characteristic were apparent when the modified INOSS or Clark case definition was applied (S6 and S7 Tables).

**Fig 2:**
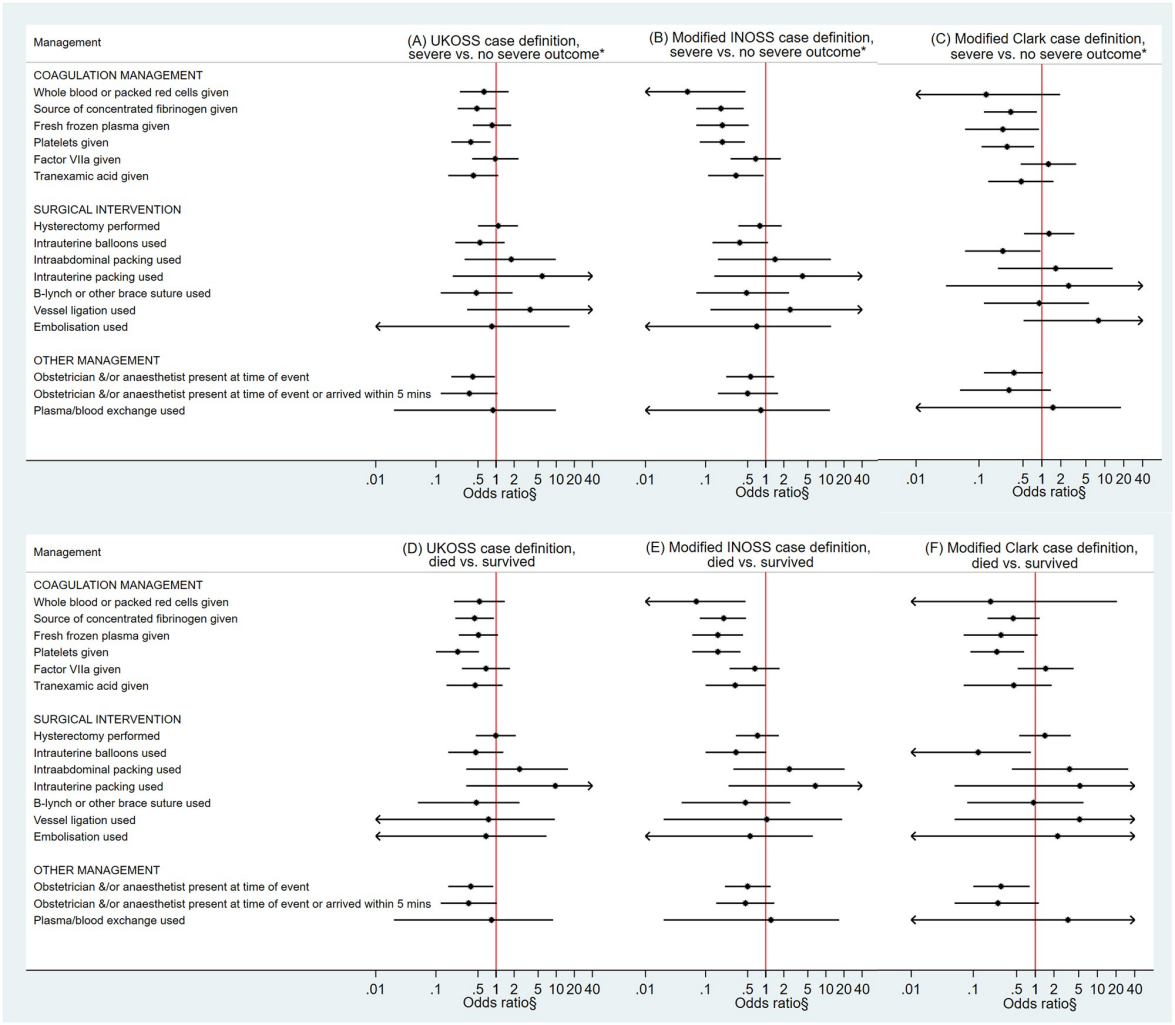
Comparison of the management of AFE cases that had severe outcome to those that did not have severe outcome using (A) UKOSS, (B) modified INOSS, and (C) modified Clark case definition and comparison of the management of AFE cases who died to those who survived using (D) UKOSS, (E) modified INOSS, and (F) modified Clark case definition. § = adjusted for cardiac arrest at presentation. * = died or had permanent neurological injury. AFE, amniotic fluid embolism; INOSS, International Network of Obstetric Survey Systems; UKOSS, UK Obstetric Surveillance System.

In terms of management and applying the UKOSS case definition, women who had a severe outcome were less likely than those who did not have a severe outcome to receive a source of concentrated fibrinogen or platelets, having adjusted for cardiac arrest at presentation (Table 5, Fig 2A). They were also less likely to have an obstetrician and/or anaesthetist present at the time of the AFE event, having adjusted for cardiac arrest at presentation. Of the women who had a hysterectomy and had data available on when the hysterectomy was done, those who had a severe outcome also had a shorter interval between the AFE event and when the hysterectomy was performed (median interval 78 minutes, IQR 55–205 minutes) compared to those who did not have a severe outcome (median interval 213 minutes, IQR 121–299 minutes). Every minute increase in interval between the AFE event and when the hysterectomy was performed was associated with a 0.6% reduction in the odds of having a severe outcome (OR 0.994, 95% CI 0.988–1.000, *p* = 0.042). However, after adjusting for cardiac arrest at presentation, this association was no longer statistically significant (aOR 0.996, 95% CI 1.000–1.002, *p* = 0.157). No other significant differences in management, including in terms of median units of blood products received or timings of when interventions were used, were found between these groups of women. When the analysis was limited to comparing women with AFE who died with those who survived, the same findings were found with respect to fibrinogen, platelets, presence of an obstetrician and/or anaesthetist (Table 5, Fig 2D), and the timing of when hysterectomy was performed (median interval 58 minutes, IQR 28–163 minutes versus 175 minutes, IQR 95–270 minutes, aOR 1.000, 95% CI 1.000–1.000, *p* = 0.747 for every minute increase in interval having adjusted for cardiac arrest at presentation). Additionally, of those given tranexamic acid, the women who died received a lower dose of this intervention compared to those who survived (median dose 0.7 g, IQR 0.4–1 g versus 2 g, IQR 1–2, *p* = 0.035). Few women received a source of concentrated fibrinogen in addition to platelets and tranexamic acid (S1 Fig).

**Table 5:** Using the UKOSS case definition, comparison of the management of AFE cases who died to those who survived and of AFE cases that had severe outcome to those that did not have severe outcome.

	No. (%)^a^ of Cases that Died (*n* = 45)	No. (%)^a^ of Cases that Survived (*n* = 171)	Unadjusted OR (95% CI, *p*-Value)	aOR (95% CI, *p*-Value)^Ϯ^	No. (%)^a^ of Cases that Had Severe Outcome^b^ (*n* = 57)^c^	No. (%)^a^ of Cases that Did Not Have Severe Outcome^b^ (*n* = 136)^c^	Unadjusted OR (95% CI, *p*-Value)	aOR (95% CI, *p*-Value)^Ϯ^
**Coagulation management**								
Whole blood or packed red cells given^¥^	36 (80)	143 (85)	0.73 (0.31–1.69, 0.458)	0.53 (0.20–1.40, 0.201)	46 (81)	111 (83)	0.87 (0.39–1.92, 0.724)	0.63 (0.25–1.62, 0.339)
Source of concentrated fibrinogen given	18 (40)	95 (56)	0.53 (0.27–1.04, 0.065)	**0.44 (0.21–0.92, 0.029)**	23 (40)	72 (53)	0.60 (0.32–1.13, 0.112)	**0.48 (0.23–0.99, 0.047)**
Fresh-frozen plasma given	26 (58)	116 (68)	0.65 (0.33–1.27, 0.208)	0.51 (0.24–1.08, 0.078)	36 (63)	86 (63)	1.00 (0.52–1.89, 0.992)	0.86 (0.41–1.79, 0.688)
Platelets given^¥^	11 (24)	84 (49)	**0.33 (0.16–0.70, 0.004)**	**0.23 (0.10–0.52, <0.001)**	18 (32)	64 (47)	**0.51 (0.27–0.98, 0.044)**	**0.38 (0.18–0.81, 0.012)**
Factor VIIa given^¥^	8 (18)	33 (20)	0.92 (0.39–2.15, 0.840)	0.68 (0.27–1.70, 0.408)	11 (20)	24 (18)	1.13 (0.51–2.50, 0.762)	0.97 (0.40–2.37, 0.953)
Tranexamic acid given^¥^	5 (12)	38 (22)	0.45 (0.17–1.23, 0.121)	0.45 (0.15–1.28, 0.133)	7 (13)	34 (25)	0.44 (0.18–1.06, 0.067)	0.42 (0.16–1.09, 0.075)
**Surgical intervention**								
Hysterectomy performed	15 (33)	45 (26)	1.40 (0.69–2.84, 0.351)	0.99 (0.46–2.13, 0.971)	19 (33)	33 (24)	1.56 (0.79–3.07, 0.197)	1.08 (0.50–2.31, 0.849)
Intrauterine balloons used^¥^	5 (12)	35 (21)	0.50 (0.18–1.37, 0.181)	0.46 (0.16–1.32, 0.147)	8 (15)	30 (22)	0.60 (0.26–1.41, 0.242)	0.54 (0.21–1.39, 0.205)
Intraabdominal packing used^¥^	3 (7)	8 (5)	1.51 (0.25–6.64, 0.784)	2.46 (0.32–15.72, 0.477)	4 (7)	8 (6)	1.25 (0.26–4.93, 0.943)	1.79 (0.30–9.84, 0.677)
Intrauterine packing used^¥^	2 (5)	1 (1)	8.45 (0.43–509.10, 0.198)	9.67 (0.32–924.66, 0.285)	2 (4)	1 (1)	2.44 (0.28–326.87, 0.363)	5.85 (0.19–564.72, 0.518)
B-lynch or other brace suture used^¥^	2 (5)	17 (10)	0.44 (0.05–1.97, 0.430)	0.47 (0.05–2.45, 0.565)	3 (5)	15 (11)	0.47 (0.13–1.66, 0.242)	0.47 (0.12–1.88, 0.284)
Vessel ligation used^¥^	1 (2)	5 (3)	0.78 (0.02–7.26, 1.000)	0.75 (0.01–9.51, 1.000)	3 (5)	3 (2)	2.54 (0.33–19.61, 0.462)	3.69 (0.33–43.68, 0.402)
Embolisation used^¥^	0 (0)	4 (2)	0.77 (0.00–6.27, 0.829)	0.68 (0.00–6.90, 0.761)	1 (2)	3 (2)	0.88 (0.02–11.32, 1.000)	0.85 (0.01–16.65, 1.000)
**Other management**								
Obstetrician and/or anaesthetist present at time of event^¥^	23 (61)	112 (75)	0.52 (0.25–1.10, 0.087)	**0.38 (0.16–0.90, 0.027)**	29 (62)	88 (73)	0.59 (0.29–1.20, 0.142)	**0.41 (0.18–0.96, 0.041)**
Obstetrician and/or anaesthetist present at time of event or arrived within 5 minutes^¥^	31 (79)	134 (87)	0.58 (0.23–1.43, 0.237)	0.35 (0.12–1.03, 0.057)	39 (80)	105 (86)	0.63 (0.27–1.50, 0.297)	0.36 (0.12–1.06, 0.064)
Plasma/blood exchange used^¥^	1 (3)	10 (6)	0.40 (0.01–2.94, 0.653)	0.84 (0.02–8.90, 1.000)	1 (2)	8 (6)	0.32 (0.01–2.48, 0.476)	0.89 (0.02–9.88, 1.000)

^a^Percentage of those with complete data.

^b^Died or had permanent neurological injury.

^c^Data on maternal morbidity only collected from 2014 in Australia.

^Ϯ^Adjusted for cardiac arrest at presentation.

^¥^Missing data/data not collected: whole blood or packed red cells *n* = 2, 0.9% in died versus survived and *n* = 2, 1.0% in severe outcome versus no severe outcome analysis; platelets *n* = 1, 0.5%; factor VIIa *n* = 3, 1.4% in died versus survived and *n* = 2, 1.0% in severe outcome versus no severe outcome analysis; tranexamic acid *n* = 4, 1.9% in died versus survived and *n* = 2, 1.0% in severe outcome versus no severe outcome analysis; intrauterine balloons *n* = 4, 1.9% in died versus survived and *n* = 2, 1.0% in severe outcome versus no severe outcome analysis; intra-abdominal packing *n* = 4, 1.9% in died versus survived and *n* = 2, 1.0% in severe outcome versus no severe outcome analysis; intrauterine packing *n* = 12, 5.5% in died versus survived and *n* = 10, 5.2% in severe outcome versus no severe outcome analysis (not collected in France); B-lynch or other brace suture *n* = 4, 1.9% in died versus survived and *n* = 2, 1.0% in severe outcome versus no severe outcome analysis; vessel ligation *n* = 4, 1.9% in died versus survived and *n* = 2, 1.0% in severe outcome versus no severe outcome analysis; embolisation *n* = 12, 5.5% in died versus survived and *n* = 10, 5.2% in severe outcome versus no severe outcome analysis (not collected in France); obstetrician and/or anaesthetist present at time of event *n* = 28, 12.9% in died versus survived and *n* = 26, 13.5% in severe outcome versus no severe outcome analysis (not collected in France); obstetrician and/or anaesthetist present at time of event or arrived within 5 minutes *n* = 23, 10.6% in died versus survived and *n* = 22, 11.4% in severe outcome versus no severe outcome analysis (not collected in France); plasma/blood exchange *n* = 12, 5.5% in died versus survived and *n* = 11, 5.7% in severe outcome versus no severe outcome analysis (not collected in France).

Bold text indicates statistically significant findings at the 5% level.

**Abbreviations**: AFE, amniotic fluid embolism; aOR, adjusted OR; CI, confidence interval; OR, odds ratio; UKOSS, UK Obstetric Surveillance System.

When the modified INOSS case definition was used, women who had a severe outcome were found to be less likely than those who did not have a severe outcome to receive whole or packed red cells, a source of concentrated fibrinogen, fresh-frozen plasma, platelets, or tranexamic acid, even having adjusted for cardiac arrest at presentation (S8 Table, Fig 2B). Of the women who received whole or packed red cells or a source of concentrated fibrinogen or had a hysterectomy and had data available on when these interventions were used, those who had a severe outcome also had a shorter interval between the AFE event and when the red cells were given (median interval 24 minutes, IQR 15–43 minutes versus 49 minutes, IQR 40–73 minutes, *p* = 0.035) or the source of concentrated fibrinogen was given (median interval 64 minutes, IQR 64–112 minutes versus 120 minutes, IQR 75–190 minutes, *p* = 0.044) or the hysterectomy was performed (median interval 78 minutes, IQR 55–205 versus 200 minutes, IQR 121–270 minutes, *p* = 0.023). However, these findings regarding intervention timings are based on low numbers of women, and the low number of outcome events in these analyses precluded adjustment for cardiac arrest at presentation, with the exception of the analysis of severe outcome in relation to interval between the AFE event and when the hysterectomy was performed. Having adjusted for cardiac arrest at presentation, this interval was not significantly different between the women who had and did not have a severe outcome (aOR 0.994 per minute increase in interval, 95% CI 0.988–1.001, *p* = 0.097). Limiting the analysis to comparing women with AFE who died to those who survived, the same findings were found (S8 Table, Fig 2E) except with respect to timing of when red blood cells or fibrinogen was given, which were not significantly different. The dose of tranexamic acid given was also found to be lower in women who died compared to those that survived (median dose 0.7 g, IQR 0.4–1 g versus 2 g, IQR 1–2, *p* = 0.0435).

Applying the modified Clark case definition, women who had a severe outcome were less likely than those who did not have a severe outcome to receive a source of concentrated fibrinogen, fresh-frozen plasma, or platelets and were less likely to have an intrauterine balloon used, having adjusted for cardiac arrest at presentation (S9 Table, Fig 2C). Of the women who received a source of concentrated fibrinogen or had tranexamic acid given and had data available on when these interventions were used, those who had a severe outcome also had a shorter interval between the AFE event and when the source of concentrated fibrinogen was given (median interval 64 minutes, IQR 40–88 minutes versus 113 minutes, IQR 75–158 minutes, *p* = 0.025) or the tranexamic acid was given (median interval 15 minutes, IQR 14–42 minutes versus 66 minutes, IQR 51–95 minutes, *p* = 0.018). However, these findings regarding intervention timings are again based on low numbers of women, and the low number of outcome events in these analyses precluded adjustment for cardiac arrest at presentation. When the analysis was limited to comparing women with AFE who died to those who survived, the women who died were less likely to have had platelets given or an intrauterine balloon used and were less likely to have had an obstetrician and/or anaesthetist present at the time of the AFE event, even having adjusted for cardiac arrest at presentation (S9 Table, Fig 2F). No other significant differences in management were apparent.

## Discussion

### Main findings

Applying different case definitions changed the estimated incidence of AFE and the estimated proportion of women with AFE who experienced poor outcomes but did not materially alter findings regarding factors associated with the occurrence of AFE and factors associated with poor maternal outcomes amongst women with AFE. Older maternal age, multiple pregnancy, polyhydramnios, placenta praevia, and induction of labour were consistently associated with the occurrence of AFE, whichever case definition was used. Additionally, giving birth by cesarean section was consistently associated with postnatal occurrence of AFE. There was also evidence that placental abruption and instrumental vaginal birth are risk factors for the condition, although these associations did not reach statistical significance when the modified Clark case definition was used, noting the more limited power of the analysis. Among women with AFE, irrespective of which case definition was used, there was evidence that those with poor maternal outcome were more likely to present with cardiac arrest and less likely to have platelets or a source of concentrated fibrinogen given, having taken into account the severity of presentation. There was also some evidence, when two out of the three case definitions were applied and the severity of presentation adjusted for when possible, that those with poor maternal outcome were less likely to have received fresh-frozen plasma, had a lower dose of tranexamic acid, and were less likely to have had an obstetrician and/or anaesthetist present at the time of the AFE event. Having adjusted for the severity of presentation, evidence of an association between poor maternal outcome and less use of whole or packed red cells, tranexamic acid, and intrauterine balloons only reached statistical significance when one of the three case definitions were applied. There was also some evidence that those with poor maternal outcome had a shorter interval between the AFE event and when certain coagulation or blood products were used, but it is important to highlight that it was not possible to adjust these findings for the severity of presentation.

### Strengths and limitations

Major strengths of this study are its multicountry population-based design with data obtained using comparable methodologies with agreed definitions and cross-checking against case criteria. In conditions such as AFE, where the rarity and rapidity of onset of the condition make randomised trials almost impossible, such international population-based studies provide the most robust level of evidence available. The UK AFE data up to January 2014 have previously been published, but this study adds data from a further four countries and includes UK data collected up to January 2018. It also adds to previous research in this field by exploring the impact of using different case definitions.

Although our study represents one of the largest studies of AFE using published case criteria, the number of women with a poor outcome was still relatively small. This was particularly so when the modified Clark case definition was applied, limiting the power of this analysis and the ability to adjust for cardiac arrest as a marker of the severity of presentation in some of the analyses. It should also be highlighted that even when the analysis was adjusted for cardiac arrest, the majority of women who experienced a poor outcome presented with this feature. Relying on voluntary reporting of cases, there is also a possibility that we have underascertained cases or ascertained only the more severe cases. However, we feel that significant underreporting is unlikely to have occurred because all of the participating countries used an active surveillance system to ascertain cases, with several reporting clinicians in each hospital. In addition, in France, the completeness of case identification was checked through the review of delivery logbooks, hospital discharge data, and laboratory files. Furthermore, most of the countries were able to cross-check their maternal deaths with another data source, as detailed in the methods. Although mortality cases were confirmed with national registry data, we recognise that retrospective review of mortality cases may falsely include cases defined as AFE as well as falsely exclude cases. However, all cases included in our study met the case definitions of AFE detailed in the methods. There nevertheless remains a small possibility that some fatal cases may have been excluded by national mortality review committees.

When studying risk factors for AFE and factors associated with poor maternal outcomes in cases of AFE, we were not able to investigate the role of factors not collected in a comparable way between countries, most notably ethnicity and socioeconomic status. Nevertheless, we were still able to perform a detailed assessment of risk factors for AFE and investigate the association between a wide range of factors and maternal outcome. However, like other observational studies, we cannot rule out the possibility of residual confounding or confounding by indication. The study used data from high-income countries, and therefore, the results are likely to be only generalizable to countries with similar resource settings.

### Interpretation

Of the women in our study who had AFE according to the UKOSS case definition, around two-thirds had AFE according to the modified INOSS case definition, and less than half had AFE according to the modified Clark case definition. A lack of evidence of coagulopathy/DIC was the most common reason for the UKOSS cases not meeting the other case definitions. As recognised by those who proposed the Clark case definition, while their criteria may result in a ‘cleaner’ set of cases for research purposes, it may exclude atypical cases [34]. Our study suggests that while the use of different case definitions affects the estimated incidence of AFE and the estimated proportion of women with AFE who experience poor outcome, it has little impact on risk factor findings or the identification of factors associated with poor maternal outcome.

In previous research, older maternal age and induction of labour are the only factors that have been identified as being consistently associated with the occurrence of AFE across five countries, although the associations did not reach statistical significance in one country [9]. The limited studies that have assessed placental abnormalities as a risk factor also suggest that placenta praevia and placental abruption are associated with the occurrence of AFE, and there is some evidence that instrumental vaginal and cesarean birth are risk factors [2,9,35]. Obtaining reliable information on factors associated with AFE has previously been hampered by not only the rarity and the difficulty in defining the condition but also the lack of consistency in the methodology used by studies. Previous data have also frequently been limited by a lack of information on the timing of AFE in relation to giving birth, resulting in uncertainty regarding whether factors such as instrumental or cesarean birth are a consequence or a cause of AFE. As well as having information on timing, our study represents, to the best of our knowledge, the first study to have pooled population-based international data on AFE obtained using consistent methodologies with agreed definitions and cross-checking against case criteria. It thus is able to provide a clearer more robust picture of the factors associated with the occurrence of AFE, although we cannot rule out the possibility of residual confounding or confounding by indication.

Limited studies have examined factors associated with severe outcomes amongst women with AFE. A study of 227 cases ascertained from a coded national hospital admissions database and a study of just 20 cases ascertained from a regional database with additional criteria to exclude false positive cases both found no significant association between various sociodemographic or obstetric-related characteristics and fatality [9,36]. A study that reviewed 44 case reports of AFE found that treatment with factor VIIa was associated with an increased risk of death or permanent disability, while a recent study that reviewed 177 case reports of AFE only found a significant association between oxytocin use during labour and fatality amongst typical cases of AFE defined as having the classical triad of symptoms (cardiac arrest/cardiovascular collapse, respiratory failure, coagulopathy) [37,38]. Another study of 180 cases identified from a coded hospital admissions population database found a crude association between induction of labour and fatality [9]. More recently, a study of 54 cases of AFE with coagulopathy reported that transfusion with a fresh-frozen plasma/red blood cell ratio of 1 or more was associated with higher survival rates [39]. Based on an analysis of the first 120 cases in the UK, included within the cases in this study, we previously reported that women who died or had permanent neurological injury were more likely to present with cardiac arrest, be from black or other minority ethnic groups, have a hysterectomy, had a shorter time interval between the AFE event and when the hysterectomy was performed, and were less likely to receive cryoprecipitate [1].

The present study was able to investigate the association between a wide range of factors and poor maternal outcome, adjusting this time for the severity of presentation when appropriate. The slight variation in findings when the different case definitions were applied is likely due to varying statistical power between the analyses. Also of note, when a significant association was found with one case definition but not another, the estimated measures of association were in the same direction and usually of a similar magnitude. Our findings may reflect a lack of time in the sickest of women to give coagulation and blood products or a lack of time to give enough of a product in the case of tranexamic acid. However, they could also indicate that better correction of coagulopathy through the use of coagulation and blood products in sufficient doses could improve maternal outcome. Potential harm from tranexamic acid is thought to be low, with the recent Woman trial finding that 1 g of intravenous tranexamic acid (with a second dose of 1 g if bleeding continued after 30 minutes or restarted within 24 hours of the first dose) reduces death due to bleeding in women with postpartum haemorrhage with no evidence of adverse effects [40]. Platelets, fresh-frozen plasma, and cryoprecipitate, on the other hand, have been associated with a number of adverse effects, including transfusion-transmitted infections, transfusion-related acute lung injury, and allergic/anaphylactic reactions [41,42]. However, the consideration of side effects becomes relative in the context of potentially life-saving therapy. Our data also suggest that having an obstetrician and/or anaesthetic present at the time of the AFE may improve maternal outcome.

### Conclusions

While the use of different case definitions of AFE influences incidence estimates and the estimated proportion of women with AFE who experience poor outcomes, it has little impact on risk factor findings or the identification of factors associated with poor maternal outcome. This study suggests that when an AFE is suspected, initial supportive obstetric care is important, but having an obstetrician and/or anaesthetist present at the time of the AFE event and use of interventions to correct coagulopathy, including the administration of an adequate dose of tranexamic acid, may be important to improve maternal outcome. Future research should focus on early detection of the coagulation deficiencies seen in AFE alongside the role of tranexamic acid and other coagulopathy management strategies.

## Supporting information

S1 Fig Using UKOSS case definition, number of women with AFE given a source of concentrated fibrinogen, platelets, and/or tranexamic acid by whether they died or survived. AFE, amniotic fluid embolism; UKOSS, UK Obstetric Surveillance System.
(DOCX)

S1 Study Protocol (DOCX)

S1 STROBE Checklist STROBE, strengthening the reporting of observational studies in epidemiology. (DOCX)

S1 Table Comparison of characteristics of women with AFE according to modified INOSS case definition and control women in the UK and Australia. AFE, amniotic fluid embolism.
(DOCX)

S2 Table Comparison of characteristics of women with AFE according to modified Clark case definition and control women in the UK and Australia. AFE, amniotic fluid embolism.
(DOCX)

S3 Table Using the modified INOSS case definition, comparison of the presentation and haematological parameters of AFE cases who died to those who survived and of AFE cases that had severe outcome to those that did not have severe outcome. AFE, amniotic fluid embolism; INOSS, International Network of Obstetric Survey Systems.
(DOCX)

S4 Table Using the modified Clark case definition, comparison of the presentation and haematological parameters of AFE cases who died to those who survived and of AFE cases that had severe outcome to those that did not have severe outcome. AFE, amniotic fluid embolism.
(DOCX)

S5 Table Using the UKOSS case definition, comparison of sociodemographic, previous obstetric and medical history, and current pregnancy characteristics of AFE cases that died to those who survived and of AFE cases that had severe outcome compared to those that did not have severe outcome. AFE, amniotic fluid embolism; UKOSS, UK Obstetric Surveillance System.
(DOCX)

S6 Table Using the modified INOSS case definition, comparison of sociodemographic, previous obstetric and medical history, and current pregnancy characteristics of AFE cases that died to those who survived and of AFE cases that had severe outcome compared to those that did not have severe outcome. AFE, amniotic fluid embolism; INOSS, International Network of Obstetric Survey Systems.
(DOCX)

S7 Table Using the modified Clark case definition, comparison of sociodemographic, previous obstetric and medical history, and current pregnancy characteristics of AFE cases that died to those who survived and of AFE cases that had severe outcome compared to those that did not have severe outcome. AFE, amniotic fluid embolism.
(DOCX)

S8 Table Using the modified INOSS case definition, comparison of the management of AFE cases who died to those who survived and of AFE cases that had severe outcome to those that did not have severe outcome. AFE, amniotic fluid embolism; INOSS, International Network of Obstetric Survey Systems.
(DOCX)

S9 Table Using the modified Clark case definition, comparison of the management of AFE cases who died to those who survived and of AFE cases that had severe outcome to those that did not have severe outcome. AFE, amniotic fluid embolism.
(DOCX)

## Abbreviations

AFE: amniotic fluid embolism
AMOSS: Australasian Maternity Outcomes Surveillance System
aOR: adjusted odds ratio
CI: confidence interval
CMACE: Centre for Maternal and Child Enquiries
DIC: disseminated intravascular coagulation
ENCMM: National Confidential Enquiries on Maternal Mortality
EPIMOMS: Épidémiologie de la Morbidité Maternelle Sévère
INOSS: International Network of Obstetric Survey Systems
INR: International Normalized Ratio
ISTH: International Society on Thrombosis and Hemostasis
NethOSS: Netherlands Obstetric Surveillance System
OR: odds ratio
SAMM: severe acute maternal morbidity
SOSS: Slovak Obstetric Survey System
STROBE: Strengthening the Reporting of Observational Studies in Epidemiology
UKOSS: UK Obstetric Surveillance System
