# Supplementary material for: Risk factors, management, and outcomes of amniotic fluid embolism: A multicountry, population-based cohort and nested case-control study
Source: PLoS Med. 2019 Nov 12;16(11):e1002962. doi: 10.1371/journal.pmed.1002962 (PMC6850527; doi:10.1371/journal.pmed.1002962)
Supplement: S1 Fig — AFE, amniotic fluid embolism; UKOSS, UK Obstetric Surveillance System. (DOCX) [file pmed.1002962.s001.docx]

**No source of concentrated fibrinogen, platelets or tranexamic acid given**

n=22 died

n=59 survived

n=1 died

n=7 survived

n=8 died

n=47 survived

n=2 died

n=22 survived

n=0 died

n=6 survived

**S1 Fig. Using UKOSS case definition, number of women with AFE given a source of concentrated fibrinogen, platelets and/or tranexamic acid by whether they died or survived.**

Diagram does not show one woman with missing data on platelets (this woman survived and known to have had a source of concentrated fibrinogen and tranexamic acid given) and 4 women with missing data on tranexamic acid (2 of these women survived, 1 of whom known to have had a source of concentrated fibrinogen and platelets given and one of whom known not to have had either of these treatments; 2 of the woman died, both of whom known to have not had a source of concentrated fibrinogen or platelets given).
