# Supplementary material for: Risk factors, management, and outcomes of amniotic fluid embolism: A multicountry, population-based cohort and nested case-control study
Source: PLoS Med. 2019 Nov 12;16(11):e1002962. doi: 10.1371/journal.pmed.1002962 (PMC6850527; doi:10.1371/journal.pmed.1002962)
Supplement: S1 Study Protocol — (DOCX) [file pmed.1002962.s002.docx]

**Study protocol: Multi-country study of the risk factors, Management and Outcomes of Amniotic-Fluid Embolism**

**1. Background**

Amniotic fluid embolism (AFE) although rare occurring in an estimated 1.7 per 100,000 maternities in the UK^1^, remains one of the leading causes of direct maternal mortality in high income countries^2-4^. AFE is characterised by unexplained sudden cardiovascular collapse, respiratory distress and disseminated intravascular coagulation. The rarity of AFE together with the fact that clinical diagnosis of the condition is one of exclusion makes it difficult to obtain reliable information concerning risk factors, management and outcomes. Previous reviews^5 6^ have highlighted the lack of consistency in the factors reported to be associated with the occurrence of AFE. Analysis of pooled international data, obtained using consistent methodologies with agreed definitions could provide more reliable information on these associated factors and hence provide the potential to develop appropriate preventative strategies. Very limited data also exists on the factors associated with severe outcomes in order to guide best practice. We recently published a study using the UK Obstetric surveillance system (UKOSS)^1^ which, despite being one of the largest conducted of AFE using validated case criteria, had limited statistical power to examine factors associated with severe outcomes. Also, despite being the most comprehensive study to date to have examined the relationship between a broad range of factors and outcomes, our study was limited in not having collected information on the amount and timing of particular management strategies used including the amount and timing of coagulation products given, so were unable to investigate this in relation to outcomes.

**2. Aims**

To use the International Network of Obstetric Survey Systems (INOSS) to pool data on cases of AFE and comparison women from multiple countries that have comparable surveillance systems to UKOSS:

1. To further investigate the risk factors, management and associated outcomes of AFE
2. To identify whether there are specific factors, such as the amount and timing of coagulation products given, that are associated with severe outcomes

**3. Methods**

We are using UKOSS to continue to identify and collect anonymised information on cases of AFE in the UK. We also propose to use INOSS to pool anonymised data on cases of AFE and comparison women from multiple countries that have comparable methodologies.

**3.1 Data collection**

*-UKOSS*

UKOSS is a system that was set up in 2005 to investigate rare and severe complications in pregnancy and childbirth in the UK. The UKOSS methodology has been described in detail elsewhere^7^. Briefly, nominated clinicians in every obstetrician-led maternity unit in the UK are sent a monthly case notification card with a list of conditions under surveillance. Upon reporting a case, clinicians are asked to complete a data collection form to confirm the diagnosis and ascertain further information concerning potential risk factors, management and outcomes. All data requested is anonymous. Since 1^st^ February 2005, clinicians have been asked to report cases of AFE. Information on comparison women is also available, identified by UKOSS reporting clinicians as the two women delivering in the same hospital immediately before other UKOSS study cases^8-14^. We are continuing to collect information on AFE using UKOSS, with a slight revision to the data collection form to include more information concerning the management of AFE. This will not only enable us to further investigate the risk factors, management and outcomes of AFE in the UK, but importantly will improve our ability to investigate whether there are specific factors, including the amount and timing of coagulation products given, that are associated with severe outcomes.

*-INOSS*

INOSS is a multi-country collaboration which was formed to facilitate studies of uncommon and severe complications in pregnancy and childbirth. Current member countries include Australia, Austria, Belgium, Denmark, Finland, France, Germany, Iceland, Italy, the Netherlands, New Zealand, Norway, Portugal, Slovakia, Spain, Sweden and the UK. As well as the data on AFE from the UK, using surveillance systems comparable to UKOSS, data on AFE has been collected in Australia and New Zealand, France, the Netherlands, Denmark and Slovakia. Information on comparison women is also available in some of these countries. Data on AFE cases and comparison women (where available) from these countries will be pooled for the purposes of this study, but all data used in this study will remain the intellectual property of their respective providing institutions. The data/information requested for this study is listed in Appendix 1.

**3.2 Monitoring ascertainment**

*-UKOSS*

Maternal deaths from AFE reported through UKOSS will be compared to information about maternal deaths from AFE obtained from the National Maternal, Newborn and Infant Clinical Outcome Review Programme run by MBBRACE-UK. If additional cases are identified through MBBRACE-UK, we will ask relevant UKOSS reporting clinician’s to complete a data collection form for the case.

*-INOSS*

Some member countries have very complete medical birth and hospital discharge registers allowing case ascertainment from their more detailed specific data collections systems to be checked.

**3.3 Case definition**

In a Delphi-study performed by INOSS, AFE was defined as “an acute cardio-respiratory collapse within 6 hours after labour, birth or ruptured membranes, with no other identifiable cause, followed by acute coagulopathy in those women who survive the initial event”. Case definitions will be compared between INOSS participating countries with the aim of identifying a uniform definition that can be applied across countries.

**3.4 Study size**

By the end of the proposed study period, we anticipate we will have data on an estimated 290 cases in total including an estimated 70 with severe outcome. This should give us 80% power at the 5% level of significance to detect ORs of 2.3 or greater and 4.0 or greater when investigating the association between specific factors and severe outcome, assuming the specific factors have a prevalence of 40% and 5% respectively.

**3.5 Statistical procedures**

-*Data considerations*

A data dictionary and data collection form will be required from each participating country. Initially, the data will be compared between countries to examine inconsistencies in coding. A common coding will be administered across datasets to ensure comparability.

-*Analysis*

All statistical analysis will be conducted in Stata 13 software. A descriptive analysis of the characteristics, management and outcomes of cases of AFE will be conducted. Putative risk factors for AFE, identified from the literature, will be evaluated by comparing women with AFE to control women using unconditional logistic regression to estimate odds ratios (ORs) and 95% CIs. A full regression model will be developed by including both explanatory and potential confounding factors in a core model if there is a preexisting hypothesis or evidence to suggest they are causally related to AFE. Addition of interaction terms to the full model and subsequent likelihood ratio testing will be used to assess plausible interactions. The management of women with AFE will be described and factors associated with severe outcomes amongst AFE cases will be investigated using unconditional logistic regression or the ӽ^2^ test, Fisher’s exact test, or Wilcoxon rank sum test, as appropriate.

# 3.6 Approvals

This non-interventional (descriptive) study will analyse anonymous data. The management of each woman participating will not be altered in any way by participation in the study. No names, addresses, dates of birth, hospital or NHS numbers were collected in order that none of the participants are individually identifiable.

Each national study has already obtained ethics committee approval to collect anonymised data. However, this study is being conducted at the NPEU, University of Oxford, UK and ethics approval may be required to export the data for analysis in the UK, depending on local protocols, which should be sought by the individual country teams. Data sharing agreement forms will be signed with each participating institution.

**3.7 Data transfer, storage, access and record retention**

The data will be transferred to researchers in the University of Oxford using the University of Oxford Oxfile secure transfer service at the institution. The data will be encrypted before being deposited into this transfer service at the University of Oxford. The password for the file will be sent separately to the researchers at the University of Oxford. From here a single copy of the file will be stored on the secure server at the National Perinatal Epidemiology Unit, University of Oxford (NPEU).

The data will be stored in a secured project folder located on a NPEU, University of Oxford storage system managed by the Information Technology (IT) team. Access to the secured project folder will be limited to the named investigators. Access to the project folder is controlled by the NPEU IT team. The storage network is protected by firewalls and intrusion prevention systems managed centrally by the IT team. The NPEU network is part of the University of Oxford internal network. The storage system will snapshot the archive regularly to prevent against accidental deletion, and backups that are undertaken are located in a secured location. No copy of the data will be made and the data will not be stored or transferred using portable data devices, for example, USB drives, CDs and DVDs.

Only named researchers will have access to the data. The NPEU maintains a comprehensive information security protocol that aims to ensure that confidentiality, integrity and availability of information is maintained at all times.

The transferred data will be disposed of 2 years after publication of the findings, in order to allow for any questions raised following publication to be addressed. All electronic files will be permanently deleted from the computer server and the research data management systems at the NPEU, University of Oxford will ensure disposal in a timely fashion.

**4. Dissemination and publication**

It is important that the clinicians who collected the data receive feedback of the outcomes of the study. The findings will be presented at specialist conferences, such as the British Maternal and Fetal Medicine Society and the Annual Scientific Meeting of the Obstetric Anaesthetists Association. The findings of this study will also be submitted for publication in a peer-reviewed journal such as the British Journal of Obstetrics and Gynaecology. The NPEU reports directly to the UK Department of Health and has a distinguished record for influencing health policy both in the UK and worldwide.

**References**

1. Fitzpatrick KE**,** Tuffnell D, Kurinczuk JJ, Knight M. Incidence, Risk factors, Management and Outcomes of Amniotic-Fluid Embolism: a population-based cohort and nested case-control study. *Bjog* 2016; 123(1): 100-9.

2. Berg CJ, Callaghan WM, Syverson C, Henderson Z. Pregnancy-related mortality in the United States, 1998 to 2005. *Obstet Gynecol* 2010;116(6):1302-9.

3. Cantwell R, Clutton-Brock T, Cooper G, Dawson A, Drife J, Garrod D, et al. Saving Mothers' Lives: Reviewing maternal deaths to make motherhood safer: 2006-2008. The Eighth Report of the Confidential Enquiries into Maternal Deaths in the United Kingdom. *Bjog* 2011;118 Suppl1:1-203.

4. Conde-Agudelo A, Romero R. Amniotic fluid embolism: an evidence-based review. *Am J Obstet Gynecol* 2009;201(5):445 e1-13.

5. Conde-Agudelo A, Romero R. Amniotic fluid embolism: an evidence-based review. *Am J Obstet Gynecol* 2009;201(5):445 e1-13.

6. Knight M, Berg C, Brocklehurst P, Kramer M, Lewis G, Oats J, et al. Amniotic fluid embolism incidence, risk factors and outcomes: a review and recommendations. *BMC pregnancy and childbirth* 2012;12:7.

7. Knight M, Kurinczuk JJ, Tuffnell D, Brocklehurst P. The UK Obstetric Surveillance System for rare disorders of pregnancy. *Bjog* 2005;112(3):263-5.

8. Knight M. Antenatal pulmonary embolism: risk factors, management and outcomes. *Bjog* 2008;115(4):453-61.

9. Knight M, Kurinczuk JJ, Spark P, Brocklehurst P. Cesarean delivery and peripartum hysterectomy. *Obstet*

*Gynecol* 2008;111(1):97-105.

10. Scott CA, Bewley S, Rudd A, Spark P, Kurinczuk JJ, Brocklehurst P, et al. Incidence, risk factors, management,

and outcomes of stroke in pregnancy. *Obstet Gynecol* 2012;120(2 Pt 1):318-24.

11. Fitzpatrick KE, Sellers S, Spark P, Kurinczuk JJ, Brocklehurst P, Knight M. Incidence and Risk Factors for

Placenta Accreta/Increta/Percreta in the UK: A National Case-Control Study. *PLoS One* 2012;7(12):e52893.

12. Fitzpatrick KE, Hinshaw K, Kurinczuk JJ, Knight M. Risk factors, management, and outcomes of hemolysis,

elevated liver enzymes, and low platelets syndrome and elevated liver enzymes, low platelets syndrome. *Obstet*

*Gynecol* 2014;123(3):618-27.

13. Knight M, Kurinczuk JJ, Spark P, Brocklehurst P. Extreme obesity in pregnancy in the United Kingdom. *Obstet*

*Gynecol* 2010;115(5):989-97.

14. Knight M. Eclampsia in the United Kingdom 2005. *Bjog* 2007;114(9):1072-8.

**Appendix 1.** **Data/information Requested**

1. Case definition
2. Questionnaire/data collection form for cases and comparison women (where available)
3. See below for desired variables and coding for case and comparison women (where available). Alternatively, if you would find it easier for us to extract this data from your dataset, please send us all the data you have collected.

| Unique case/comparison woman identifier |  |
| --- | --- |
| Case or comparison woman | Binary:  1 = case  0 = control |
| ***Sociodemographic characteristics*** |  |
| Maternal age | Continuous |
| Ethnicity | Describe |
| Socio-economic status | Describe |
| Height at booking | Continuous |
| Weight at booking | Continuous |
| Body Mass index at booking (kg/m^2^) | Continuous |
| Smoking status | Describe |
| ***Previous obstetric & medical history*** |  |
| Parity | Continuous. Describe definition used e.g. Number of completed pregnancies ≥24 weeks prior to this current pregnancy |
| History of allergy | Binary:  1 = Yes  2 = No |
| History of atopy (asthma, eczema, hayfever) | Binary:  1 = Yes  2 = No |
| Essential hypertension | Binary:  1 = Yes  2 = No |
| Diabetes mellitus | Binary:  1 = Yes  2 = No |
| Other Pre-existing medical conditions/problems | Describe |
| ***Current pregnancy*** |  |
| Final estimated date of delivery (EDD) | dd/mm/yy |
| Multiple pregnancy | Binary:  1 = Yes  2 = No |
| Placenta praevia diagnosed | Binary:  1 = Yes  2 = No  and if yes, state grade of placenta praevia |
| Placental abruption | Binary:  1 = Yes  2 = No |
| Any hypertensive disorder | Binary:  1 = Yes  2 = No |
| Pregnancy induced hypertension | Binary:  1 = Yes  2 = No  and if yes date of onset (dd/mm/yy) |
| Pre-eclampsia (hypertension and proteinuria) | Binary:  1 = Yes  2 = No  and if yes date of onset (dd/mm/yy) |
| Eclampsia | Binary:  1 = Yes  2 = No  and if yes:  date of onset (dd/mm/yy)  time of onset (hh:mm) |
| Other hypertensive disorder | Describe |
| Chorioamnionitis | Binary:  1 = Yes  2 = No |
| Polyhydramnios | Binary:  1 = Yes  2 = No |
| Gestational diabetes | Binary:  1 = Yes  2 = No |
| Other problems in this pregnancy | Binary:  1 = Yes  2 = No  and if yes describe |
| ***Diagnosis of Amniotic-fluid embolism (only case women)*** |  |
| Whether any of following features present at or immediately preceding diagnosis:  -Acute fetal compromise  -Cardiac arrest  -Cardiac rhythm problems  -Coagulopathy  -Hypotension  -Maternal haemorrhage  -Premonitory symptoms e.g. restless, agitation, numbness, tingling  -Seizure  -Shortness of breath | For each feature -  Binary:  1 = present  2 = Not present  Rant features in order of occurrence (1,2,3 etc) |
| Lab tests:  -Hb g/dL  -Platelet count (x10^9^/L)  -PT (sec)  -INR  -APPT (sec)  -APTT (ratio) APTT  -Fibrinogen (g/dL)  -D-dimer (ng/ml)  -Tryptase (μg/l) | First results after diagnosis and the worst haematological parameters recorded at the time of the AFE or indicate if not recorded |
| **Maternal event (only case women)** |  |
| Date and time of event | dd/mm/yy hh:mm |
| Gestational age at time of event | Weeks |
| Date and time diagnosis first considered | dd/mm/yy hh:mm |
| Membranes ruptured at time of event | Binary:  1 = Yes  2 = No  and if yes:  date and time of rupture dd/mm/yy hh:mm  was rupture 1 = artificial or 2 = spontaneous |
| Meconium staining of liquor | Categorical  1 = fresh  2 = old  3 = none |
| Fetal distress before maternal collapse | Binary:  1 = Yes  2 = No  and if yes specify/describe |
| At the time of the event was the woman not in labour, first stage, second stage or post-delivery | Categorical  1 = Not in labour  2 = First stage  3 = Second stage  4 = Post-delivery |
| Contraction frequency at time of event (number in 10 minutes) | Continuous |
| Any anaesthetic/analgesia at time of collapse | Categorical  1 = spinal  2 = epidural  3 = combined spinal epidural  4 = GA  5 = none |
| Consultant obstetrician present at time of collapse | Binary:  1 = Yes  2 = No |
| Date and time Consultant obstetrician first saw woman after collapse | dd/mm/yy hh:mm or state if did not see woman |
| Consultant anaesthetist present at time of collapse | Binary:  1 = Yes  2 = No |
| Date and time anaesthetist first saw woman after collapse | dd/mm/yy hh:mm or state if did not see woman |
| Senior midwife present at time of collapse | Binary:  1 = Yes  2 = No |
| Date and time Senior midwife first saw woman after collapse | dd/mm/yy hh:mm or state if did not see woman |
| ***Management (only case women)*** |  |
| Treated with/given Syntocinon infusion | Binary:  1 = Yes  2 = No  and if yes:  date first given (dd/mm/yy)  time first given (hh:mm)  total dose and units |
| Treated with/given Ergometrine | Binary:  1 = Yes  2 = No  and if yes:  date first given (dd/mm/yy)  time first given (hh:mm)  total dose and units |
| Treated with/given Prostaglandin F2α | Binary:  1 = Yes  2 = No  and if yes:  date first given (dd/mm/yy)  time first given (hh:mm)  total dose and units |
| Treated with/given Misoprostol | Binary:  1 = Yes  2 = No  and if yes:  date first given (dd/mm/yy)  time first given (hh:mm)  total dose and units |
| Treated with/given Hemabate | Binary:  1 = Yes  2 = No  and if yes:  date first given (dd/mm/yy)  time first given (hh:mm)  total dose and units |
| Treated with/given Whole blood or packed red cells | Binary:  1 = Yes  2 = No  and if yes:  date first given (dd/mm/yy)  time first given (hh:mm)  total dose and units |
| Treated with/given Cryoprecipitate | Binary:  1 = Yes  2 = No  and if yes:  date first given (dd/mm/yy)  time first given (hh:mm)  total dose and units |
| Treated with/given Fresh frozen plasma | Binary:  1 = Yes  2 = No  and if yes:  date first given (dd/mm/yy)  time first given (hh:mm)  total dose and units |
| Treated with/given Platelets | Binary:  1 = Yes  2 = No  and if yes:  date first given (dd/mm/yy)  time first given (hh:mm)  total dose and units |
| Treated with/given Cell salvage | Binary:  1 = Yes  2 = No  and if yes:  date first given (dd/mm/yy)  time first given (hh:mm)  total dose and units |
| Treated with/given Fibrinogen | Binary:  1 = Yes  2 = No  and if yes:  date first given (dd/mm/yy)  time first given (hh:mm)  total dose and units |
| Treated with/given Factor VIIa | Binary:  1 = Yes  2 = No  and if yes:  date first given (dd/mm/yy)  time first given (hh:mm)  total dose and units |
| Treated with/given Heparin | Binary:  1 = Yes  2 = No  and if yes:  date first given (dd/mm/yy)  time first given (hh:mm)  total dose and units |
| Treated with/given tranexamic acid | Binary:  1 = Yes  2 = No  and if yes:  date first given (dd/mm/yy)  time first given (hh:mm)  total dose and units |
| Other treatments given | Binary:  1 = Yes  2 = No  and if yes:  Specify/describe  date first given (dd/mm/yy)  time first given (hh:mm)  total dose and units |
| Treated with intrauterine balloons | Binary:  1 = Yes  2 = No  and date (dd/mm/yy)  and time (hh:mm) |
| Treated with intrauterine packing | Binary:  1 = Yes  2 = No  and if yes:  date (dd/mm/yy)  time (hh:mm) |
| Treated with B-lynch or other brace suture | Binary:  1 = Yes  2 = No  and if yes:  date (dd/mm/yy)  time (hh:mm) |
| Treated with vessel embolism | Binary:  1 = Yes  2 = No  and if yes:  date (dd/mm/yy)  time (hh:mm) |
| Treated with vessel ligation | Binary:  1 = Yes  2 = No  and if yes:  date (dd/mm/yy)  time (hh:mm) |
| Treated with intra-arterial balloons | Binary:  1 = Yes  2 = No  and if yes:  date (dd/mm/yy)  time (hh:mm) |
| Treated with hysterectomy | Binary:  1 = Yes  2 = No  and if yes:  date (dd/mm/yy)  time (hh:mm) |
| Treated with intra-abdominal packing | Binary:  1 = Yes  2 = No  and if yes:  date (dd/mm/yy)  time (hh:mm) |
| Treated with exchange transfusion | Binary:  1 = Yes  2 = No  and if yes:  date (dd/mm/yy)  time (hh:mm) |
| Treated with plasma exchange | Binary:  1 = Yes  2 = No  and if yes:  date (dd/mm/yy)  time (hh:mm) |
| Treated with apheresis | Binary:  1 = Yes  2 = No  and if yes:  date (dd/mm/yy)  time (hh:mm) |
| ***Delivery*** |  |
| Delivery induced | Binary:  1 = Yes  2 = No  and if yes:  reason for induction (describe)  was vaginal prostaglandin used (1 = Yes, 2 = no)  Describe preparation and total dose of prostaglandin given (mg) |
| Woman labour | Binary:  1 = Yes  2 = No  And if yes:  Date labour diagnosed (dd/mm/yy)  time labour diagnosed (hh:mm)  Used syntocinon during labour (1 = Yes, 2 = no)  Duration of syntocinon during labour  Hyperstimulation occur – contractions more than 5 in 10 minutes (1 = Yes, 2 = no)  How long did hyperstimulation occur (hh:mm) |
| Delivered by caesarean section | Binary:  1 = Yes  2 = No  and if yes:  whether elective or emergency (1 = elective, 2 = emergency)  Describe indication for caesarean section  Method of anaesthesia (1 = regional, 2 = GA) |
| Manual removal of placenta | Binary:  1 = Yes  2 = No |
| Gestational age at delivery | Weeks |
| ***Outcomes - woman*** |  |
| Woman admitted to ITU/HDU | Binary:  1 = Yes  2 = No  And if yes:  Date of admission (dd/mm/yy)  Time of admission (hh:mm)  Duration of stay (days) or indicate if woman still in ITU/HDU or transferred to another hospital |
| Did woman have permanent neurological injury (e.g. hypoxic brain injury, persistent vegetative state) | Binary:  1 = Yes  2 = No  And if yes give details |
| Did the woman have disseminated intravascular coagulopathy (DIC) | Binary:  1 = Yes  2 = No |
| Any other major maternal morbidity occur | Binary:  1 = Yes  2 = No  And if yes give details |
| Did woman die | Binary:  1 = Yes  2 = No  And if yes:  Date of death (dd/mm/yy)  Time of death (hh:mm)  State primary cause of death as stated in death certificate (or state if not known)  Was post-mortem examination undertaken (1 = yes, 2 = no)  Were fetal squames or hair found in lungs (1 = yes, 2 = no) |
| ***Outcomes – infant (if more than one infant please provide for each infant)*** |  |
| Date and time of delivery | dd/mm/yy hh:mm |
| Mode of delivery | Describe including whether women had an instrumental vaginal delivery (ventouse or forceps), non-instrumental vaginal delivery, pre-labour caesarean or caesarean section after onset of labour |
| Birthweight | Continuous in g |
| Sex of infant | Male = 1  Female = 2  Indeterminate = 3 |
| Infant stillborn | Binary:  1 = Yes  2 = No  And please given definition of stillborn used and if yes:  Whether stillborn antepartum or intapartum (1 = antepartum, 2 = intrapartum) |
| 5 minute Apgar | Continuous |
| Infant admitted to neonatal unit | Binary:  1 = Yes  2 = No  And if yes:  Duration of stay (days) or indicate if infant still in neonatal unit or if infant transferred to another hospital |
| Any other major infant complications occur | Binary:  1 = Yes  2 = No  And if yes give details |
| Did infant die | Binary:  1 = Yes  2 = No  And if yes:  Date of death (dd/mm/yy)  Primary cause of death as stated on death certificate (or state if not known) |

Any additional information you may have collected which would help to identify whether case meets INOSS Delphi definition or Clark et al’s proposed definition of AFE:

-***INOSS Delphi definition***: An acute cardio-respiratory collapse within 6 hours after labour, birth or ruptured membranes, with no other identifiable cause, followed by acute coagulopathy in those women who survive the initial event

-***Clark definition***:

1. Sudden onset of cardiorespiratory arrest, or both hypotension (systolic blood pressure < 90mm Hg) and respiratory compromise (dyspnea, cyanosis, or peripheral capillary oxygen saturation <90%

2. Documentation of DIC following appearance of these initial signs or symptoms, using scoring system of Scientific and Standardization Committee on DIC of the ISTH, modified for pregnancy* (prior to loss of sufficient blood to itself account for dilutional or shock-related consumptive coagulopathy)

3. Clinical onset during labour or within 30 min of delivery of placenta

4. No fever (≥38.0^o^C) during labour

*Modified International Society on Thrombosis and Hemostasis scoring system for overt disseminated intravascular coagulation in pregnancy:

- Platelet count: >100,000/mL = 0, <100,000/mL = 1, <50,000/mL = 2

- Prolonged prothrombin time or international normalized ratio: <25% increase = 0, 25-50% increase = 1, >50% increase = 2

- Fibrinogen level: >200 mg/L = 0, <200 mg/L = 1

Score ≥3 is compatible with overt disseminated intravascular coagulation in pregnancy
