# Supplementary material for: Risk factors, management, and outcomes of amniotic fluid embolism: A multicountry, population-based cohort and nested case-control study
Source: PLoS Med. 2019 Nov 12;16(11):e1002962. doi: 10.1371/journal.pmed.1002962 (PMC6850527; doi:10.1371/journal.pmed.1002962)
Supplement: S1 Table — AFE, amniotic fluid embolism. (DOCX) [file pmed.1002962.s004.docx]

**S1 Table. Comparison of characteristics of women with AFE according to modified INOSS case definition and control women in the UK and Australia**

|  | **Number (%)^a^ of cases (n=131)** | **Number (%)^a^ of controls (n=4938)** | **Unadjusted OR (95% CI, P-value)** | **Adjusted OR (95% CI, P-value)**Ϯ |
| --- | --- | --- | --- | --- |
| **Sociodemographic characteristics** |  |  |  |  |
| Maternal age (years)¥ |  |  |  |  |
| Less than 35 | 72 (55) | 3884 (79) | 1 | 1 |
| 35 or more | 59 (45) | 1048 (21) | **3.04 (2.14-4.31, <0.001)** | **2.37 (1.57-3.59, <0.001)** |
| Body mass index at booking (kg/m2)¥ |  |  |  |  |
| Less than 30 | 96 (78) | 3677 (80) | 1 | 1 |
| 30 or more | 27 (22) | 892 (20) | 1.16 (0.75-1.79, 0.503) | 1.04 (0.63-1.71, 0.877) |
| Smoking status¥ |  |  |  |  |
| Never/ex smoker | 115 (89) | 3979 (82) | 1 | 1 |
| Smoked during pregnancy | 14 (11) | 860 (18) | **0.56 (0.32-0.99, 0.044)** | 0.92 (0.49-1.72, 0.794) |
| **Previous obstetric and medical history** |  |  |  |  |
| Parity¥^b^ |  |  |  |  |
| 0 | 49 (38) | 2134 (43) | 1 | 1 |
| 1 or more | 80 (62) | 2797 (57) | 1.25 (0.87-1.79, 0.232) | 1.01 (0.67-1.52, 0.978) |
| Chronic hypertension¥ |  |  |  |  |
| No | 125 (97) | 4862 (99) | 1 | 1 |
| Yes | 4 (3) | 48 (1) | 3.24 (0.84--9.05, 0.086) | 0.48 (0.09-2.48, 0.381) |
| Pre-existing diabetes¥ |  |  |  |  |
| No | 128 (98) | 4860 (99) | 1 |  |
| Yes | 2 (2) | 51 (1) | 1.49 (0.17-5.77, 0.798) |  |
| **Current pregnancy characteristics** |  |  |  |  |
| Multiple pregnancy |  |  |  |  |
| No | 120 (92) | 4866 (99) | 1 | 1 |
| Yes | 11 (8) | 71 (1) | **6.28 (2.92-12.-32, <0.001)** | **5.39 (2.42-12.01, <0.001)** |
| Gestational diabetes¥ |  |  |  |  |
| No | 120 (92) | 4742 (97) | 1 | 1 |
| Yes | 10 (8) | 160 (3) | **2.47 (1.13-4.82, 0.024)** | 1.70 (0.82-3.54, 0.153) |
| Hypertensive disorder¥ |  |  |  |  |
| No | 114 (88) | 4688 (96) | 1 | 1 |
| Yes | 16 (12) | 215 (4) | **3.06 (1.78-5.26, <0.001)** | 1.58 (0.78-3.20, 0.201) |
| Polyhydramnios¥ |  |  |  |  |
| No | 122 (94) | 4866 (99) | 1 | 1 |
| Yes | 8 (6) | 32 (1) | **9.96 (3.88-22.65, <0.001)** | **7.40 (2.71-20.21, <0.001)** |
| Placenta praevia¥ |  |  |  |  |
| No | 115 (88) | 4878 (99) | 1 | 1 |
| Yes | 15 (12) | 31 (1) | **20.47 (9.98-40.36, <0.001)** | **22.49 (10.41-48.57, <0.001)** |
| Placental abruption¥ |  |  |  |  |
| No | 128 (98) | 4893 (100) | 1 | 1 |
| Yes | 2 (2) | 8 (0.2) | 9.54 (0.98-48.48, 0.052) | **6.11 (1.02-36.49, 0.047)** |
| Induction of labor using any method^c^¥ |  |  |  |  |
| No | 67 (51) | 3752 (76) | 1 | 1 |
| Yes | 64 (49) | 1179 (24) | **3.04 (2.14-4.31, <0.001)** | **3.67 (2.42-5.57, <0.001)** |
| Gestational age at delivery (weeks)^d^¥ |  |  |  |  |
| Term (37-41) | 102 (78) | 4412 (90) | 1 | 1 |
| Pre-term (<37) | 26 (20) | 369 (8) | **3.05 (1.88-4.80, <0.001)** | 1.70 (0.93-3.09, 0.083) |
| Post term (42 or more) | 2 (2) | 136 (3) | 0.64 (0.08-2.40, 0.796) | 0.54 (0.13-2.28, 0.402) |
| Macrosomia (birthweight of 4000g or more)^d^¥ |  |  |  |  |
| No | 116 (91) | 4359 (89) | 1 | 1 |
| Yes | 11 (9) | 558 (11) | 0.74 (0.40-1.38, 0.346) | 0.72 (0.36-1.42, 0.234) |

^a^ Percentage of those with complete data

ϮAdjusted for all variables in table apart from pre-existing diabetes

^b^ Australia (AMOSS): number of previous pregnancies ≥20 wks gestation or resulting in birth of a baby weighting ≥400g; UK (UKOSS): number of completed pregnancies ≥ 24 wks gestation

^c^ In Australia, data on induction of labor only collected for women who labored. Women who did not labor in Australia assumed to have had no induction of labor.

^d^ excludes 10 women who had pregnancies ending before 24 weeks gestation

¥Missing data: maternal age n=6, 0.1%; body mass index n=377, 7.4%; smoking status n=101, 2.0%; parity n=9, 0.2%; chronic hypertension n=30, 0.6%; pre-existing diabetes n=28, 0.6%; multiple pregnancy n=1, 0.02%; gestational diabetes n=37, 0.7%; hypertensive disorder n=36, 0.7%; polyhydramnios n=41, 0.8%; placenta praevia n=30, 0.6%; placental abruption n=38, 0.7%; labor induced n=7, 0.1%; gestational age at delivery n=12, 0.2%; macrosomia n=15, 0.3%

Bold text indicates statistically significant findings at the 5% level
