# Supplementary material for: Risk factors, management, and outcomes of amniotic fluid embolism: A multicountry, population-based cohort and nested case-control study
Source: PLoS Med. 2019 Nov 12;16(11):e1002962. doi: 10.1371/journal.pmed.1002962 (PMC6850527; doi:10.1371/journal.pmed.1002962)
Supplement: S2 Table — AFE, amniotic fluid embolism. (DOCX) [file pmed.1002962.s005.docx]

**S2 Table. Comparison of characteristics of women with AFE according to modified Clark case definition and control women in the UK and Australia**

|  | **Number (%)^a^ of cases (n=92)** | **Number (%)^a^ of controls (n=4938)** | **Unadjusted OR (95% CI, P-value)** | **Adjusted OR (95% CI, P-value)**Ϯ |
| --- | --- | --- | --- | --- |
| **Sociodemographic characteristics** |  |  |  |  |
| Maternal age (years)¥ |  |  |  |  |
| Less than 35 | 50 (54) | 3884 (79) | 1 | 1 |
| 35 or more | 42 (46) | 1048 (21) | **3.11 (2.05-4.72, <0.001)** | **2.39 (1.46-3.90, <0.001)** |
| Body mass index at booking (kg/m2)¥ |  |  |  |  |
| Less than 30 | 67 (77) | 3677 (80) | 1 | 1 |
| 30 or more | 20 (23) | 892 (20) | 1.23 (0.74-2.04, 0.421) | 1.08 (0.61-1.94, 0.788) |
| Smoking status¥ |  |  |  |  |
| Never/ex smoker | 78 (87) | 3979 (82) | 1 | 1 |
| Smoked during pregnancy | 12 (13) | 860 (18) | 0.71 (0.39-1.31, 0.277) | 1.27 (0.65-2.50, 0.485) |
| **Previous obstetric and medical history** |  |  |  |  |
| Parity¥^b^ |  |  |  |  |
| 0 | 35 (38) | 2134 (43) | 1 | 1 |
| 1 or more | 556 (61) | 2797 (57) | 1.20 (0.78-1.84, 0.406) | 1.01 (0.62-1.64, 0.977) |
| Chronic hypertension¥ |  |  |  |  |
| No | 88 (97) | 4862 (99) | 1 | 1 |
| Yes | 3 (3) | 48 (1) | 3.45 (0.67-11.04, 0.130) | 0.72 (0.13-4.10, 0.711) |
| Pre-existing diabetes¥ |  |  |  |  |
| No | 90 (99) | 4860 (99) | 1 |  |
| Yes | 1 (1) | 51 (1) | 1.06 (0.03-6.32, 1.000) |  |
| **Current pregnancy characteristics** |  |  |  |  |
| Multiple pregnancy |  |  |  |  |
| No | 84 (91) | 4866 (99) | 1 | 1 |
| Yes | 8 (9) | 71 (1) | **6.52 (2.63-14.14, <0.001)** | **6.49 (2.63-15.98, <0.001)** |
| Gestational diabetes¥ |  |  |  |  |
| No | 83 (90) | 4742 (97) | 1 | 1 |
| Yes | 9 (10) | 160 (3) | **3.21 (1.39-6.55, 0.007)** | **2.22 (1.00-4.91, 0.049)** |
| Hypertensive disorder¥ |  |  |  |  |
| No | 85 (92) | 4688 (96) | 1 | 1 |
| Yes | 7 (8) | 215 (4) | 1.80 (0.69-3.93, 0.227) | 1.15 (0.45-2.90, 0.772) |
| Polyhydramnios¥ |  |  |  |  |
| No | 84 (91) | 4866 (99) | 1 | 1 |
| Yes | 8 (9) | 32 (1) | **14.45 (5.58-33.27, <0.001)** | **10.59 (3.79-29.61, <0.001)** |
| Placenta praevia¥ |  |  |  |  |
| No | 78 (85) | 4878 (99) | 1 | 1 |
| Yes | 14 (15) | 31 (1) | **28.15 (13.29-57.07, <0.001)** | **31.30 (13.68-71.62, <0.001)** |
| Placental abruption¥ |  |  |  |  |
| No | 91 (99) | 4893 (100) | 1 | 1 |
| Yes | 1 (1) | 8 (0.2) | 6.71 (0.15-50.96, 0.309) | 4.70 (0.46-47.59, 0.190) |
| Induction of labor using any method^c^¥ |  |  |  |  |
| No | -49 (53) | 3752 (76) | 1 | 1 |
| Yes | 43 (47) | 1179 (24) | **2.79 (1.84-4.23, <0.001)** | **3.87 (2.35-6.36, <0.001)** |
| Gestational age at delivery (weeks)^d^¥ |  |  |  |  |
| Term (37-41) | 70 (76) | 4412 (90) | 1 | 1 |
| Pre-term (<37) | 20 (22) | 369 (8) | **3.41 (1.94-5.76, <0.001)** | 1.77 (0.88-3.54, 0.109) |
| Post term (42 or more) | 2 (2) | 136 (3) | 0.93 (0.11-3.54, 1.000) | 0.79 (0.18-3.37, 0.748) |
| Macrosomia (birthweight of 4000g or more)^d^¥ |  |  |  |  |
| No | 82 (91) | 4359 (89) | 1 | 1 |
| Yes | 8 (9) | 558 (11) | 0.76 (0.37-1.58, 0.467) | 0.85 (0.39-1.86, 0.690) |

^a^ Percentage of those with complete data

ϮAdjusted for all variables in table apart from pre-existing diabetes

^b^ Australia (AMOSS): number of previous pregnancies ≥20 wks gestation or resulting in birth of a baby weighting ≥400g; UK (UKOSS): number of completed pregnancies ≥ 24 wks gestation

^c^ In Australia, data on induction of labor only collected for women who labored. Women who did not labor in Australia assumed to have had no induction of labor.

^d^ excludes 10 women who had pregnancies ending before 24 weeks gestation

¥Missing data: maternal age n=6, 0.1%; body mass index n=374, 7.4%; smoking status n=101, 2.0%; parity n=9, 0.2%; chronic hypertension n=29, 0.6%; pre-existing diabetes n=28, 0.6%; multiple pregnancy n=1, 0.02%; gestational diabetes n=36, 0.7%; hypertensive disorder n=35, 0.7%; polyhydramnios n=40, 0.8%; placenta praevia n=29, 0.6%; placental abruption n=37, 0.7%; labor induced n=7, 0.1%; gestational age at delivery n=11, 0.2%; macrosomia n=13, 0.3%

Bold text indicates statistically significant findings at the 5% level
