# Supplementary material for: Risk factors, management, and outcomes of amniotic fluid embolism: A multicountry, population-based cohort and nested case-control study
Source: PLoS Med. 2019 Nov 12;16(11):e1002962. doi: 10.1371/journal.pmed.1002962 (PMC6850527; doi:10.1371/journal.pmed.1002962)
Supplement: S3 Table — AFE, amniotic fluid embolism; INOSS, International Network of Obstetric Survey Systems. (DOCX) [file pmed.1002962.s006.docx]

**S3 Table. Using modified INOSS case definition, comparison of the presentation and haematological parameters of AFE cases who died to those who survived and of AFE cases that had severe outcome to those that did not have severe outcome**

|  | **No. (%)^a^ of cases that died (n=42)** | **No. (%)^a^ of cases that survived (n=100)** | **Unadjusted OR (95% CI, P-value)** | **Adjusted OR (95% CI, P-value)Ϯ** | **No. (%)^a^ of cases that had severe outcome^b^ (n=51)^c^** | **No. (%)^a^ of cases that did not have severe outcome^b^ (n=74)^c^** | **Unadjusted OR (95% CI, P-value)** | **Adjusted OR (95% CI, P-value)Ϯ** |
| --- | --- | --- | --- | --- | --- | --- | --- | --- |
| **When AFE occurred¥** |  |  |  |  |  |  |  |  |
| Before or at delivery | 23 (56) | 53 (53) | 1 |  | 27 (54) | 43 (58) | 1 |  |
| Post delivery | 18 (44) | 47 (47) | 0.88 (0.42-1.83, 0.738) |  | 23 (46) | 31 (42) | 1.18 (0.57-2.43, 0.651) |  |
| **Features at presentation**‡ |  |  |  |  |  |  |  |  |
| Acute fetal compromise | 14 (33) | 39 (39) | 0.78 (0.37-1.67, 0.525) |  | 18 (35) | 31 (42) | 0.76 (0.36-1.58, 0.458) |  |
| Cardiac arrest | 38 (90) | 52 (52) | **8.77 (2.91-26.41, <0.001)** | **5.33 (1.61-17.57, 0.006)** | 46 (90) | 36 (49) | **9.71 (3.47-27.18, <0.001)** | **7.06 (2.30-21.63, 0.001)** |
| Cardiac rhythm problems¥ | 9 (25) | 31 (32) | 0.71 (0.30-1.69, 0.438) |  | 11 (25) | 23 (32) | 0.72 (0.31-1.68, 0.454) |  |
| Coagulopathy | 24 (57) | 88 (88) | **0.18 (0.08-0.43, <0.001)** | **0.16 (0.04-0.64, 0.009)** | 31 (61) | 65 (88) | **0.21 (0.09-0.53, <0.001)** | 0.28 (0.07-1.05, 0.060) |
| Hypotension¥ | 19 (46) | 82 (82) | **0.19 (0.09-0.42, <0.001)** | 0.41 (0.16-1.02, 0.055) | 25 (50) | 61 (82) | **0.21 (0.09-0.48, <0.001)** | 0.52 (0.20-1.34, 0.174) |
| Maternal haemorrhage | 28 (67) | 83 (83) | **0.41 (0.18-0.94, 0.034)** | 1.84 (0.44-7.74, 0.406) | 34 (67) | 62 (84) | **0.39 (0.17-0.90, 0.029)** | 0.89 (0.22-3.53, 0.873) |
| Premonitory symptoms¥ | 19 (49) | 40 (43) | 1.24 (0.58-2.62, 0.582) |  | 23 (49) | 28 (39) | 1.51 (0.72-3.16, 0.280) |  |
| Seizures¥ | 6 (15) | 14 (14) | 1.02 (0.36-2.86, 0.976) |  | 8 (16) | 11 (15) | 1.07 (0.40-2.89, 0.888) |  |
| Shortness of breath¥ | 13 (32) | 35 (36) | 0.81 (0.37-1.76, 0.594) |  | 15 (30) | 27 (37) | 0.73 (0.34-1.58, 0.423) |  |
| **Platelet count (mL)¥** |  |  |  |  |  |  |  |  |
| More than 100,000 | 10 (31) | 22 (23) | 1 |  | 12 (29) | 14 (20) | 1 |  |
| 50-100,000 | 10 (31) | 45 (47) | 0.49 (0.18-1.35, 0.167) |  | 13 (32) | 36 (51) | 0.42 (0.16-1.14, 0.090) |  |
| Less than 50,000 | 12 (38) | 29 (30) | 0.91 (0.33-2.49, 0.855) |  | 16 (39) | 20 (29) | 0.93 (0.34-2.57, 0.894) |  |
| **INR¥** |  |  |  |  |  |  |  |  |
| <25% increase | 2 (25) | 6 (19) | 1 |  | 2 (22) | 4 (29) | 1 |  |
| 25-50% increase | 1 (13) | 6 (19) | 0.52 (0.01-12.73, 1.000) |  | 1 (11) | 3 (21) | 0.69 (0.01-20.30, 1.000) |  |
| >50% increase | 5 (63) | 19 (61) | 0.80 (0.09-10.41, 1.000) |  | 6 (67) | 7 (50) | 1.67 (0.16-24.78, 0.988) |  |
| **Fibrinogen (g/L)¥** |  |  |  |  |  |  |  |  |
| 2 or more | 0 (0) | 6 (16) | 1 |  | 1 (10) | 3 (18) | 1 |  |
| Less than 2 | 7 (100) | 31 (84) | 1.70 (0.21-infinity, 0.659) |  | 9 (90) | 14 (82) | 1.89 (0.13-112.32, 1.000) |  |

^a^ Percentage of those with complete data

^b^ Died or had permanent neurological injury

^c^ Data on maternal morbidity only collected from 2014 in Australia

Ϯ Adjusted for factors that were significant (p<0.05) in unadjusted model

‡ Features at presentation are not mutually exclusive

¥Missing data/data not collected: when AFE occurred n=1, 0% in died vs. survived & n=1, 0.8% in severe outcome vs. no severe outcome analysis; cardiac rhythm problems n=9, 6.3% in died vs. survived & n=8, 6.4% in severe outcome vs. no severe outcome analysis (not collected in France); hypotension n=1, 0.7% in died vs. survived & n=1, 0.8% in severe outcome vs. no severe outcome analysis; premonitory symptoms n=11, 7.7% in died vs. survived & n=6, 4.8% in severe outcome vs. no severe outcome analysis (not collected in France); seizures n=4, 2.8% in died vs. survived & n=2, 1.6% in severe outcome vs. no severe outcome analysis; shortness of breath n=5, 3.5% in died vs. survived & n=2, 1.6% in severe outcome vs. no severe outcome analysis; platelet count n=14, 9.9% in died vs. survived & n=14, 11.2% in severe outcome vs. no severe outcome analysis; INR n=103, 72.5% in died vs. survived & n=102, 81.6% in severe outcome vs. no severe outcome analysis (not collected in the UK before February 2015 & not collected in France); fibrinogen n=98, 69.0% in died vs. survived & n=98, 78.4% in severe outcome vs. no severe outcome analysis (not collected in the UK before February 2015)

Bold text indicates statistically significant findings at the 5% level
