# Supplementary material for: Risk factors, management, and outcomes of amniotic fluid embolism: A multicountry, population-based cohort and nested case-control study
Source: PLoS Med. 2019 Nov 12;16(11):e1002962. doi: 10.1371/journal.pmed.1002962 (PMC6850527; doi:10.1371/journal.pmed.1002962)
Supplement: S7 Table — AFE, amniotic fluid embolism. (DOCX) [file pmed.1002962.s010.docx]

**S7 Table. Using modified Clark case definition, comparison of socio-demographic, previous obstetric, medical history and current pregnancy characteristics of AFE cases that died to those who survived and of AFE cases that had severe outcome compared to those that did not have severe outcome**

|  | **No. (%)^a^ of cases that died**  **(n=24)** | **No. (%)^a^ of cases that survived (n=74)** | **Unadjusted OR (95% CI, P-value)** | **No. (%)^a^ of cases that had severe outcome^b^ (n=33)^c^** | **No. (%)^a^ of cases that did not have severe outcome^b^ (n=51)^c^** | **Unadjusted OR (95% CI, P-value)** |
| --- | --- | --- | --- | --- | --- | --- |
| **Sociodemographic characteristics** | |  |  |  |  |  |
| Maternal age (years)¥ |  |  |  |  |  |  |
| Less than 35 | 11 (46) | 41 (55) | 1 | 19 (58) | 29 (57) | 1 |
| 35 or more | 13 (54) | 33 (45) | 1.47 (0.58-3.70, 0.416) | 14 (42) | 22 (43) | 0.97 (0.40-2.35, 0.949) |
| Body mass index at booking (kg/m2)¥ |  |  |  |  |  |  |
| Less than 30 | 16 (76) | 57 (80) | 1 | 24 (80) | 37 (76) | 1 |
| 30 or more | 5 (24) | 14 (20) | 1.27 (0.31-4.50, 0.894) | 6 (20) | 12 (24) | 0.77 (0.25-2.33, 0.645) |
| Smoking status¥ |  |  |  |  |  |  |
| Never/ex smoker | 22 (96) | 61 (85) | 1 | 28 (88) | 42 (86) | 1 |
| Smoked during pregnancy | 1 (4) | 11 (15) | 0.25 (0.01-1.95, 0.311) | 4 (13) | 7 (14) | 0.86 (0.17-3.76, 1.000) |
| **Previous obstetric and medical history** | |  |  |  |  |  |
| Parity¥^d^ |  |  |  |  |  |  |
| 0 | 6 (26) | 30 (41) | 1 | 9 (28) | 24 (47) | 1 |
| 1 or more | 17 (74) | 44 (59) | 1.93 (0.68-5.47, 0.215) | 23 (72) | 27 (53) | 2.27 (0.88-5.85, 0.089) |
| Chronic hypertension¥ |  |  |  |  |  |  |
| No | 22 (92) | 72 (99) | 1 | 31 (94) | 49 (98) | 1 |
| Yes | 2 (8) | 1 (1) | 6.39 (0.32-391.34, 0.301) | 2 (6) | 1 (2) | 3.12 (0.16-190.23, 0.693) |
| Pre-existing diabetes¥ |  |  |  |  |  |  |
| No | 23 (96) | 73 (100) | 1 | 32 (97) | 50 (100) | 1 |
| Yes | 1 (4) | 0 (0) | 3.04 (0.08-infinity, 0.495) | 1 (3) | 0 (0) | 1.52 (0.04-infinity, 0.795) |
| **Current pregnancy characteristics** | |  |  |  |  |  |
| Multiple pregnancy |  |  |  |  |  |  |
| No | 23 (96) | 67 (91) | 1 | 31 (94) | 47 (92) | 1 |
| Yes | 1 (4) | 7 (9) | 0.42 (0.01-3.56, 0.741) | 2 (6) | 4 (8) | 0.76 (0.07-5.68, 1.000) |
| Gestational diabetes¥ |  |  |  |  |  |  |
| No | 20 (83) | 68 (92) | 1 | 28 (85) | 47 (92) | 1 |
| Yes | 4 (17) | 6 (8) | 2.24 (0.42-10.58, 0.405) | 5 (15) | 4 (8) | 2.08 (0.41-11.40, 0.481) |
| Hypertensive disorder¥ |  |  |  |  |  |  |
| No | 21 (88) | 69 (93) | 1 | 29 (88) | 47 (92) | 1 |
| Yes | 3 (13) | 5 (7) | 1.97 (0.43-8.95, 0.379) | 4 (12) | 4 (8) | 1.61 (0.28-9.37, 0.771) |
| Induction of labor using any method^e^¥ | |  |  |  |  |  |
| No | 10 (42) | 42 (57) | 1 | 14 (42) | 26 (51) | 1 |
| Yes | 14 (58) | 32 (43) | 1.84 (0.72-4.67, 0.201) | 19 (58) | 25 (49) | 1.41 (0.58-3.41, 0.444) |
| Prostaglandin labor induction |  |  |  |  |  |  |
| Induced without prostaglandin | 1 (7) | 2 (6) | 1 | 2 (11) | 0 (0) | 1 |
| Induced with prostaglandin | 13 (92) | 30 (94) | 0.87 (0.04-55.02, 1.000) | 17 (89) | 25 (100) | 0.30 (0-3.99, 0.362) |
| Oxytocin used during labor¥ |  |  |  |  |  |  |
| No | 9 (64) | 30 (65) | 1 | 14 (61) | 20 (65) | 1 |
| Yes | 5 (36) | 16 (35) | 1.04 (0.30-3.64, 0.949) | 9 (39) | 11 (35) | 1.17 (0.38-3.56, 0.784) |
| Mode of delivery |  |  |  |  |  |  |
| Spontaneous vaginal | 2 (8) | 5 (7) | 1 | 3 (9) | 3 (6) | 1 |
| Instrumental vaginal | 1 (4) | 11 (15) | 0.25 (0.00-2.79, 0.592) | 3 (9) | 7 (14) | 0.45 (0.03-5.51, 0.783) |
| Cesarean section | 21 (88) | 58 (78) | 0.91 (0.14-10.20, 1.000) | 27 (82) | 41 (80) | 0.66 (0.08-5.32, 0.935) |

^a^ Percentage of those with complete data

^b^ Died or had permanent neurological injury

^c^ Data on maternal morbidity only collected from 2014 in Australia

^d^ Australia (AMOSS): number of previous pregnancies ≥20 wks gestation or resulting in birth of a baby weighting ≥400g; France (EPIMOMS): number of previous completed pregnancies ≥ 22 wks gestation; Netherlands (NOSS), Slovakia (SOSS) and UK (UKOSS): number of completed pregnancies ≥ 24 wks gestation

^e^ In Australia, data on induction of labor only collected for women who labored. Women who did not labor in Australia assumed to have had no induction of labor.

¥ Missing data: body mass index n=6, 6.1% in died vs. survived & n=5, 6.0% in severe outcome vs. no severe outcome analysis; smoking status n=3, 3.1% in died vs. survived & n=3, 3.6% in severe outcome vs. no severe outcome analysis; parity n=1, 1.0% in died vs. survived & n=1, 1.2% in severe outcome vs. no severe outcome analysis; chronic hypertension n=1, 1.0% in died vs. survived & n=1, 1.2% in severe outcome vs. no severe outcome analysis; pre-existing diabetes n=1, 1.0% in died vs. survived & n=1, 1.2% in severe outcome vs. no severe outcome analysis;

Bold text indicates statistically significant findings at the 5% level
