# Supplementary material for: Risk factors, management, and outcomes of amniotic fluid embolism: A multicountry, population-based cohort and nested case-control study
Source: PLoS Med. 2019 Nov 12;16(11):e1002962. doi: 10.1371/journal.pmed.1002962 (PMC6850527; doi:10.1371/journal.pmed.1002962)
Supplement: S8 Table — AFE, amniotic fluid embolism; INOSS, International Network of Obstetric Survey Systems. (DOCX) [file pmed.1002962.s011.docx]

**S8 Table. Using modified INOSS case definition, comparison of the management of AFE cases who died to those who survived and of AFE cases that had severe outcome to those that did not have severe outcome**

|  | **No. (%)^a^ of cases that died (n=42)** | **No. (%)^a^ of cases that survived (n=100)** | **Unadjusted OR (95% CI, P-value)** | **Adjusted OR (95% CI, P-value)Ϯ** | **No. (%)^a^ of cases that had severe outcome^b^ (n=51)^c^** | **No. (%)^a^ of cases that did not have severe outcome^b^ (n=74)^c^** | **Unadjusted OR (95% CI, P-value)** | **Adjusted OR (95% CI, P-value)Ϯ** |
| --- | --- | --- | --- | --- | --- | --- | --- | --- |
| **Coagulation management** | |  |  |  |  |  |  |  |
| Whole blood or packed red cells given | 33 (79) | 98 (98) | **0.08 (0.01-0.40, <0.001)** | **0.07 (0.01-0.46, 0.002)** | 41 (80) | 73 (99) | **0.06 (0.00-0.43, 0.001)** | **0.05 (0.00-0.46, 0.002)** |
| Source of concentrated fibrinogen given | 15 (36) | 71 (71) | **0.23 (0.11-0.49, <0.001)** | **0.20 (0.08-0.47, <0.001)** | 18 (35) | 53 (72) | **0.22 (0.10-0.46, <0.001)** | **0.18 (0.07-0.43, <0.001)** |
| Fresh frozen plasma given | 23 (55) | 87 (87) | **0.18 (0.08-0.42, <0.001)** | **0.16 (0.06-0.42, <0.001)** | 30 (59) | 65 (88) | **0.20 (0.08-0.48, <0.001)** | **0.19 (0.07-0.52, 0.001)** |
| Platelets given¥ | 11 (26) | 63 (64) | **0.20 (0.09-0.45, <0.001)** | **0.16 (0.06-0.38, <0.001)** | 16 (31) | 47 (64) | **0.25 (0.12-0.54, <0.001)** | **0.19 (0.08-0.45, <0.001)** |
| Factor VIIa given¥ | 8 (20) | 23 (23) | 0.80 (0.32-1.97, 0.630) | 0.66 (0.25-1.70, 0.387) | 10 (20) | 18 (25) | 0.76 (0.32-1.83, 0.546) | 0.68 (0.26-1.78, 0.436) |
| Tranexamic acid given¥ | 4 (10) | 28 (29) | **0.28 (0.09-0.85, 0.025)** | **0.31 (0.10-0.99, 0.048)** | 6 (12) | 24 (32) | **0.29 (0.11-0.78, 0.014)** | **0.32 (0.11-0.92, 0.034)** |
| **Surgical intervention** |  |  |  |  |  |  |  |  |
| Hysterectomy performed | 14 (33) | 35 (35) | 0.93 (0.43-1.99, 0.849) | 0.73 (0.32-1.65, 0.448) | 18 (35) | 25 (34) | 1.07 (0.51-2.26, 0.861) | 0.80 (0.35-1.84, 0.605) |
| Intrauterine balloons used¥ | 4 (10) | 26 (27) | **0.31 (0.10-0.95, 0.040)** | 0.32 (0.10-1.02, 0.054) | 6 (12) | 21 (28) | **0.35 (0.13-0.95, 0.039)** | 0.37 (0.13-1.09, 0.071) |
| Intraabdominal packing used¥ | 3 (8) | 4 (4) | 1.90 (0.26-11.80, 0.656) | 2.47 (0.29-20.48, 0.540) | 3 (6) | 4 (5) | 1.14 (0.16-7.08, 1.000) | 1.43 (0.16-12.11, 1.000) |
| Intrauterine packing used¥ | 2 (5) | 1 (1) | 5.20 (0.26-313.55, 0.387) | 6.68 (0.24-569.22, 0.400) | 2 (4) | 1 (1) | 3.24 (0.16-195.70, 0.663) | 4.09 (0.14-366.15, 0.693) |
| B-lynch or other brace suture used¥ | 2 (5) | 11 (11) | 0.42 (0.04-2.06, 0.425) | 0.46 (0.04-2.57, 0.562) | 3 (6) | 9 (12) | 0.47 (0.08-2.03, 0.432) | 0.48 (0.07-2.44, 0.518) |
| Vessel ligation used¥ | 1 (3) | 3 (3) | 0.81 (0.02-10.49, 1.000) | 1.04 (0.02-18.70, 1.000) | 2 (4) | 2 (3) | 1.53 (0.11-21.73, 1.000) | 2.55 (0.12-54.54, 0.825) |
| Embolisation used¥ | 0 (0) | 3 (3) | 0.65 (0-6.15, 0.729) | 0.55 (0-6.06, 0.647) | 0 (0) | 2 (3) | 0.65 (0-8.46, 0.749) | 0.71 (0-12.23, 0.814) |
| **Other management** |  |  |  |  |  |  |  |  |
| Obstetrician &/or anaesthetist present at time of event¥ | 22 (59) | 63 (70) | 0.63 (0.28-1.39, 0.253) | 0.50 (0.21-1.21, 0.121) | 27 (61) | 45 (67) | 0.78 (0.35-1.72, 0.532) | 0.56 (0.22-1.38, 0.207) |
| Obstetrician &/or anaesthetist present at time of event or arrived within 5 mins¥ | 30 (79) | 78 (84) | 0.72 (0.28-1.88, 0.503) | 0.46 (0.15-1.39, 0.168) | 37 (80) | 56 (82) | 0.88 (0.34-2.30, 0.796) | 0.50 (0.16-1.60, 0.243) |
| Plasma/blood exchange used¥ | 1 (3) | 6 (6) | 0.41 (0.01-3.58, 0.728) | 1.22 (0.02-16.64, 1.000) | 1 (2) | 5 (7) | 0.30 (0.01-2.81, 0.486) | 0.83 (0.01-11.72, 1.000) |

^a^ Percentage of those with complete data

^b^ Died or had permanent neurological injury

^c^ Data on maternal morbidity only collected from 2014 in Australia

Ϯ Adjusted for cardiac arrest at presentation

¥Missing data/data not collected: platelets n=1, 0.7% in died vs. survived & n=1, 0.8% in severe outcome vs. no severe analysis; factor v11a n=2, 1.4% in died vs. survived & n=2, 1.6% in severe outcome vs. no severe outcome analysis; tranexamic acid n=4, 2.8% in died vs. survived & n=2, 1.6% in severe outcome vs. no severe outcome analysis; intrauterine balloons n=4, 2.8% in died vs. survived & n=2, 1.6% in severe outcome vs. no severe outcome analysis; intraabdominal packing n=4, 2.8% in died vs. survived & n=2, 1.6% in severe outcome vs. no severe outcome analysis; intrauterine packing n=8, 5.6% in died vs. survived & n=6, 4.8% in severe outcome vs. no severe outcome analysis (not collected in France); B-lynch or other brace suture n=4, 2.8% in died vs. survived & n=2, 1.6% in severe outcome vs. no severe outcome analysis; vessel ligation n=4, 2.8% in died vs. survived & n=2, 1.6% in severe outcome vs. no severe outcome analysis; embolisation n=8, 5.6% in died vs. survived & n=6, 4.8% in severe outcome vs. no severe outcome analysis (not collected in France); obstetrician &/or anaesthetist present at time of event n=15, 10.6% in died vs. survived & n=14, 11.2% in severe outcome vs. no severe outcome analysis (not collected in France); obstetrician &/or anaesthetist present at time of event or arrived within 5 mins n=11, 7.7% in died vs. survived & n=11, 8.8% in severe outcome vs. no severe outcome analysis (not collected in France); plasma/blood exchange n=7, 4.9% in died vs. survived & n=7, 5.6% in severe outcome vs. no severe outcome analysis (not collected in France)

Bold text indicates statistically significant findings at the 5% level
